# Node Distortions as a Means of Defect Engineering in Zr-Based MOFs

**DOI:** 10.1021/jacs.6c06764

**Published:** 2026-07-30

**Authors:** Till Schertenleib, Timo M. O. Felder, Nazanin Taheri, Wei Shi, Tanaporn Na Narong, Mehrdad Asgari, Pattaraphon Rodlamul, Simon J. L. Billinge, Wendy L. Queen

**Affiliations:** † Institute of Chemical Sciences and Engineering (ISIC), 27218 École Polytechnique Fédérale de Lausanne (EPFL), 1950 Sion, Switzerland; ‡ Department of Chemistry, University of Copenhagen, Copenhagen 2100, Denmark; § Research Center for Analytical Sciences, Department of Chemistry, College of Sciences, Northeastern University, Shenyang 110819, China; ∥ Department of Applied Physics and Applied Mathematics, 5798 Columbia University, New York, New York 10027, United States; ⊥ Departement of Chemical Engineering and Biotechnology, 2152 University of Cambridge, Cambridge CB3 0AS, U.K.; # Lucy Cavendish College, 2152 University of Cambridge, Lady Margaret Rd, Cambridge CB3 0BU, U.K.; ¶ Materials Department, University of California, Santa Barbara, Santa Barbara, California 93106, United States

## Abstract

Defect engineering in Zr-based metal–organic frameworks (Zr-MOFs) has focused primarily on missing-linker defects. However, recent studies suggest that node dehydroxylation–which creates distortions and coordinatively unsaturated Zr sites (Zr_cus_)–may have a more significant impact on properties. The present work uses pair distribution function (PDF) and thermogravimetric analysis coupled with systematic defect manipulation to study the effect of node dehydroxylation and missing-linker defects in UiO-66. By employing rapid heat treatment (*RHT*) under humid flow, we tracked the transition from high-symmetry [Zr_6_O_4_(OH)_4_]^12+^ to distorted [Zr_6_O_6_]^12+^ nodes. This structural evolution significantly improves As­(V) uptake, whereas increasing the number of missing linkers–via chemical treatment or *RHT* of mixed-ligand frameworks–fails to enhance performance. Crucially, our detection of distorted nodes in as-synthesized UiO-66 also raises the possibility that these defects were silently present in many earlier studies that span various applications, where their role in governing performance may have been inadvertently overlooked. The present study challenges the prevailing “missing-linker” paradigm and establishes cluster dehydroxylation as a defect-engineering strategy to enhance Lewis-acidic performance in Zr-MOFs.

## Graphical abstract

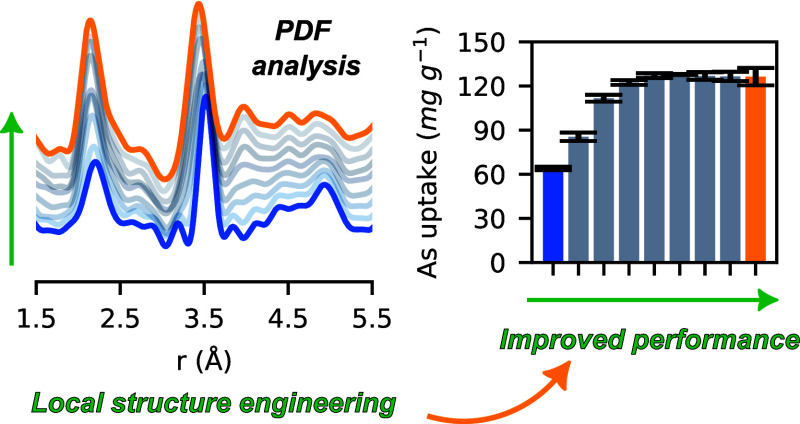

## Introduction

Zr-based metal−organic frameworks (Zr-MOFs), composed of {Zr_6_} metal nodes interlinked with organic carboxylate linkers, have emerged as one of the most extensively studied classes of MOFs, largely due to their exceptional structural versatility and chemical and thermal stability.^1–7^ The properties of Zr-MOFs are strongly determined by the reactivity/activity of Zr sites within the framework. For example, Zr-MOFs are known to have Lewis acidic metal sites, which drive chemical transformation reactions.^8^ In other applications, Zr sites are the adsorption sites for toxic pollutants in water.^9–11^ In that context, the activity of Zr-MOFs is largely determined by the coordination environment of Zr within the frameworks’ metal nodes.

The idealized canonical UiO-66 structure is composed of [Zr_6_O_4_(OH)_4_]^12+^ nodes capped by 12 terephthalate linkers (BDC^2−^). In this structure, Zr^4+^ ions are coordinatively saturated by eight ligands, including four metal−ligand (Zr–O-L) bonds and four intracluster bonds (Zr-μ_3_O–Zr and Zr-μ_3_OH–Zr). For any substrate to reach Zr, one of these ligands must be replaced by the substrate or removed prior to binding. Hence, the properties of Zr-MOFs are largely dictated by defects, as they improve the activity of Zr sites by tuning their coordination environment and/or increasing their accessibility.^12^ While defects are inherent to any crystalline material, defect engineering to tune and increase the density of active sites in Zr-MOFs is an active research field.^1,13,14^

The prevailing model for defect engineering in Zr-MOFs, like UiO-66, assumes that the core structure of the {Zr_6_} node (for example, [Zr_6_O_4_(OH)_4_]^12+^ in UiO-66), remains intact with defects described as compositional vacancy defects (missing linkers or missing clusters).^15^ One of the most widely used methods to induce such compositional defects in Zr-MOFs is by either tuning modulator concentration during synthesis,^16^ or by postsynthetic thermal activation/treatments.^13,17^ Often, little attention is given to how such defects actually affect the coordination environment of Zr sites within the metal nodes. This is problematic, because it views the MOF building blocks—particularly the {Zr_6_} nodes—as inert building blocks that do not change structurally during those manipulations. However, there is a small but growing body of research that shows how dynamic {Zr_6_} nodes are during thermal activation/treatment.^18–22^ For example, early studies have shown that during thermal activation intracluster μ_3_OH groups can be removed from the {Zr_6_} nodes, resulting in symmetry breaking and distortion of the cluster as determined via extended X-ray absorption fine structures (EXAFS).^18^ More recently, PDF analysis of UiO-66 and NU-1000 revealed irreversible node distortions during thermal treatments,^20^ and others have shown that spray-dried amorphous powders of UiO-66 contain features in their local structure attributed to the presence of dehydroxylated clusters.^23^

Importantly, unlike missing-linker defects, cluster dehydroxylation lowers the Zr coordination number to 7, creating coordinatively unsaturated Zr (Zr_cus_) sites that act as strong Lewis acids, confirmed by infrared spectroscopy.^24–26^ Missing-linker defects, on the other hand, are not known to lower Zr’s coordination number. Instead, linkers are typically replaced by other groups such as terminal H_2_O/OH^−^, Cl^−^, and F^−^ ligands or monocarboxylic acid modulators.^27–30^ Thus, one would expect that cluster dehydroxylation would have a stronger effect on the activity of Zr sites than missing-linker defects, since Lewis acids are strong binding sites or highly active catalytic sites.^31–33^ Even the earliest studies that systematically installed thermolabile ligands in UiO-66 to induce missing-linker defects attributed the improved activity and the presence of permanent Lewis acid sites, in part, to cluster dehydroxylation occurring during thermal activation.^31,34^

To date, a critical knowledge gap exists: we do not know whether the functional properties attributed to defects in Zr-MOFs arise from missing-linker defects, node dehydroxylation, or both. This ambiguity is particularly problematic because thermal treatments—commonly used to induce missing-linker defects via thermolysis—simultaneously create node distortions, which have been overlooked in previous studies. Linking these two defect types to material properties is difficult, as they typically arise together and could also influence one another. For instance, missing-linker defects have been shown to induce small distortions in {Zr_6_} nodes using density functional theory (DFT) calculations.^20^ Further, it was previously shown that the identity of ligands capping {Zr_6_} clusters, i.e., carboxylate versus terminal OH^−^/H_2_O/Cl^−^ ligands, can affect the type of node distortion induced during thermal activation, and whether they are reversible or not.^20,22^ Previous work by us has also shown that the emergence of node distortion in DUT-67 is accompanied by an increased number of linker defects.^22^

Here, we systematically investigate how the properties of Zr-MOFs correlate with missing-linker defects and {Zr_6_} node-dehydroxylation in a Zr-MOF known as UiO-66.^35^ We selected As­(V) (HAsO_4_^2−^/H_2_AsO_4_^−^) removal from water as a model application because numerous studies have attributed UiO-66’s high uptake capacity to missing-linker defects,^36–38^ making it an ideal test case. Further, As­(V) has a considerable X-ray cross-section—As has an atomic number of 33 compared to 40 of Zr—which allows us to use total scattering experiments and PDF analysis to study how As interacts with the dehydrated and the hydrated clusters. By combining rapid heat treatment (*RHT*) under humid flow^22^ with chemical post-treatments^36^ and mixed-ligand frameworks,^38^ we can vary the number and type of each defect. UiO-66 was selected over other Zr-MOFs for two key reasons. First, its high connectivity confers exceptional thermal stability, preventing framework collapse during the thermal treatments used to induce defects.^22^ Second, it readily undergoes ligand substitution with thermolabile BDC-NH_2_ linkers (2-aminoterephthalic acid), providing a handle for tuning the fraction of missing linkers after thermal treatment and thus more control over the ratio of node distortions to missing linkers defects. The present work demonstrates that the average local structure in Zr-MOFs can be gradually modified and that those changes clearly correlate with the As­(V) uptake capacity. Our results indicate that node distortions creating Zr_cus_ sites, rather than missing-linker defects, are the primary driver of improved As­(V) uptake. Furthermore, PDF analysis reveals that distorted nodes are present even in as-synthesized UiO-66, raising the question whether their contribution to performance has been overlooked—or misattributed to missing-linker defects—across the many applications for which Zr-MOFs have been studied for.

## Results and Discussion

### Local Structure Analysis of UiO-66

UiO-66 was synthesized using a previously reported method.^39^ We selected this protocol because it yields reproducible surface areas and excludes linker-competing carboxylate modulators. Protocols that use acetic acid or formic acid modulators tend to yield materials containing lower average node connectivity due to BDC^2−^ substitution with the modulator,^40^ complicating quantitative defect analysis via TGA. The as-synthesized UiO-66 powder was then exposed to *RHT* under humid airflow at 400 °C for 8 different time intervals ranging from 1 to 40 min (see Methods for details). The samples are named according to their treatment time as UiO-66-RHT-X, where X is the number of minutes of *RHT*.

UiO-66 gradually becomes amorphous, with complete loss of crystallinity after the 7 min *RHT*, as can be seen from the loss of Bragg peaks in the PXRD patterns (Figure 1a). This is also reflected by the loss of signals at distances beyond 10 Å in the PDFs (Figure 1b). Only a very broad signal remains around 11 Å, attributed to intercluster Zr–Zr distances, indicating that the clusters are still connected via organic linkers, albeit with substantial structural disorder as indicated by the broadness of the peaks. TGA indicates ∼ 13.8% of BDC^2−^ linkers are removed during 40 min of *RHT*. In absolute numbers, the number of missing linkers increases from 1.24 (parent UiO-66) to 1.90 (UiO-66-RHT-40) out of 6 ligands per {Zr_6_} node (Figure S1 and Table S1). Further, the TGA profiles of the parent UiO-66 and UiO-66-RHT-1 clearly show the significant mass loss between 200 and 300 °C, associated with cluster dehydroxylation, while the curves become almost completely flat in that temperature range after 7 min of *RHT* (Figure S1b). This indicates that the *RHT* leads to complete dehydroxylation of the sample as treatment time increases. BET surface area calculations from nitrogen adsorption isotherms reveal that the structure remains porous after *RHT* despite full amorphization. Interestingly, the BET surface area steadily decreases from 1422 *m* ^2^ *g* ^−1^ for the parent MOF to 435 *m* ^2^ *g* ^−1^ for UiO-66-RHT-7. Thereafter, the surface area plateaus with a modest drop to 421 *m* ^2^ *g* ^−1^ for UiO-66-RHT-40 (Figure S2 and Table S3). Importantly, this contrasts with our previous study on DUT-67, where prolonged *RHT* (of up to 10 min) leads to pore collapse, as denoted by a complete loss of surface area. The difference can likely be attributed to UiO-66’s greater {Zr_6_} node connectivity (12 vs 8 in DUT-67). PDF analysis reveals that the {Zr_6_} nodes in UiO-66 (Figure 1b) undergo structural transformations similar to those observed in DUT-67 under similar *RHT* conditions^22^ and in UiO-66 and NU-1000 during thermal treatment under inert atmosphere or dry air^18–20,41^

**Figure 1:**
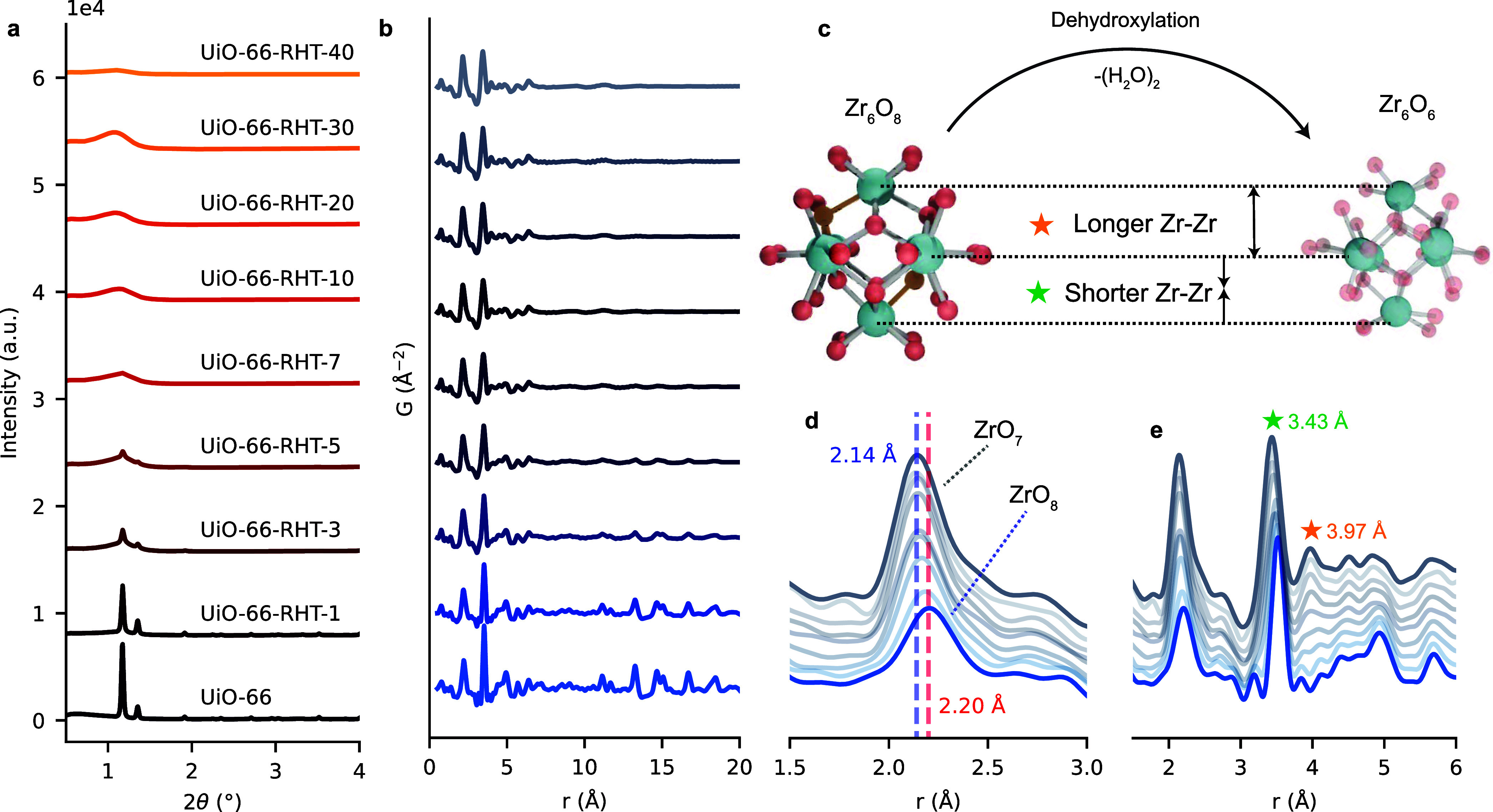
(a) Synchrotron PXRD (λ = 0.24486 Å) of as-synthesized UiO-66 (parent MOF) and samples exposed to *RHT* for time intervals ranging from 1 to 40 min. (b) Corresponding PDFs of each sample, with the same sample ordering from bottom to top as in (a). (c) Structural model of pristine (Zr_6_O_8_) and dehydroxylated (Zr_6_O_6_) Zr oxo clusters (Blue: Zr, Red: O, Orange: bridging O group removed during dehydroxylation). Dehydroxylation creates O-vacancies and Zr_cus_ sites in the cluster, causing distortions in the first and second coordination shells, corresponding to Zr–O bonds and Zr–Zr pairs, respectively. (d) The shift of the first-shell peak to lower-*r* is shown, indicating a reduced coordination number of Zr. (e) The full change in the local structure is shown, highlighting distortion in the second shell, with shortened and elongated Zr–Zr pairs in the dehydroxylated cluster compared to the pristine cluster.

Node distortions are evident in both the first (Zr–O) and second coordination shell-peaks (Zr–Zr) of the PDFs. The idealized {Zr_6_} cluster contains 6 Zr atoms arranged in an octahedral geometry with 12 edges and 8 faces. Each Zr atom is 8-coordinated with 4 metal-carboxylate bonds and 4 Zr-μ_3_O bonds bridging neighboring Zr–Zr pairs. On average, two of those bridging bonds are protonated for charge balance, yielding Zr-μ_3_OH bonds.^18^ The arrangement of the Zr atoms can be grouped into pairs of edge-sharing atoms (12 edges in total) and pairs of atoms arranged diagonally to each other (3 diagonal pairs). The PDF of the idealized octahedral {Zr_6_} cluster contains three main fingerprint features at approximately 2.2, 3.5, and 5 Å, corresponding to the Zr–O bonds and the edge-sharing and diagonal Zr–Zr pairs. These three peaks can be best seen in Figure 1e (blue curve at the bottom). The intensity of the peak in the PDF is determined by the multiplicity of the atom pairs and the scattering powers of the atoms involved. In the first shell, the average Zr–O bond length shifts from 2.20 to 2.14 Å (Figure 1d), reflecting a reduced coordination number of Zr. This change is also visible as broadening of the X-ray absorption near-edge structures (XANES) of the Zr K-edge (Figure S5). The PDF and XANES data strongly suggest that the coordination environment around the Zr sites changes: the shift of the average first-shell peak in the PDF indicates a reduced coordination number, and the broadening of the XANES suggests that the coordination sphere around the absorber becomes less centrosymmetric^22^—a result of the induced distortions in the {Zr_6_} nodes. Even greater changes are observed in the second shell, which stems from the removal of μ_3_OH groups from the faces of the {Zr_6_} octahedra, allowing the edge-sharing Zr–Zr pairs to move away from each other.^18^ This creates the splitting of the peak at 3.53 Å in UiO-66,^22^ originating from edge-sharing Zr pairs, into two peaks centered at 3.43 and 3.97 Å after 40 min of *RHT* (Figure 1e). Figure 2 describes in detail how specific features in the PDF relate to structural characteristics of the {Zr_6_} nodes. The first shell peak (Zr–O) is sensitive to changes in the oxygen-containing species coordinated to Zr, including both missing-linker defects (e.g., ligand replacement by terminal H_2_O or OH^−^) and dehydroxylation (e.g., the loss of μ_3_O or μ_3_OH species) (Figure 2a–c,h). In contrast, the second shell peak (Zr–Zr) captures node distortions within the {Zr_6_} cluster, manifesting as a splitting of the Zr–Zr distances that arise from the elongation along one direction and the contraction along another—a signature of cluster dehydroxylation and the associated O-vacancies (Figure 2d–g,i).^18,22^

**Figure 2:**
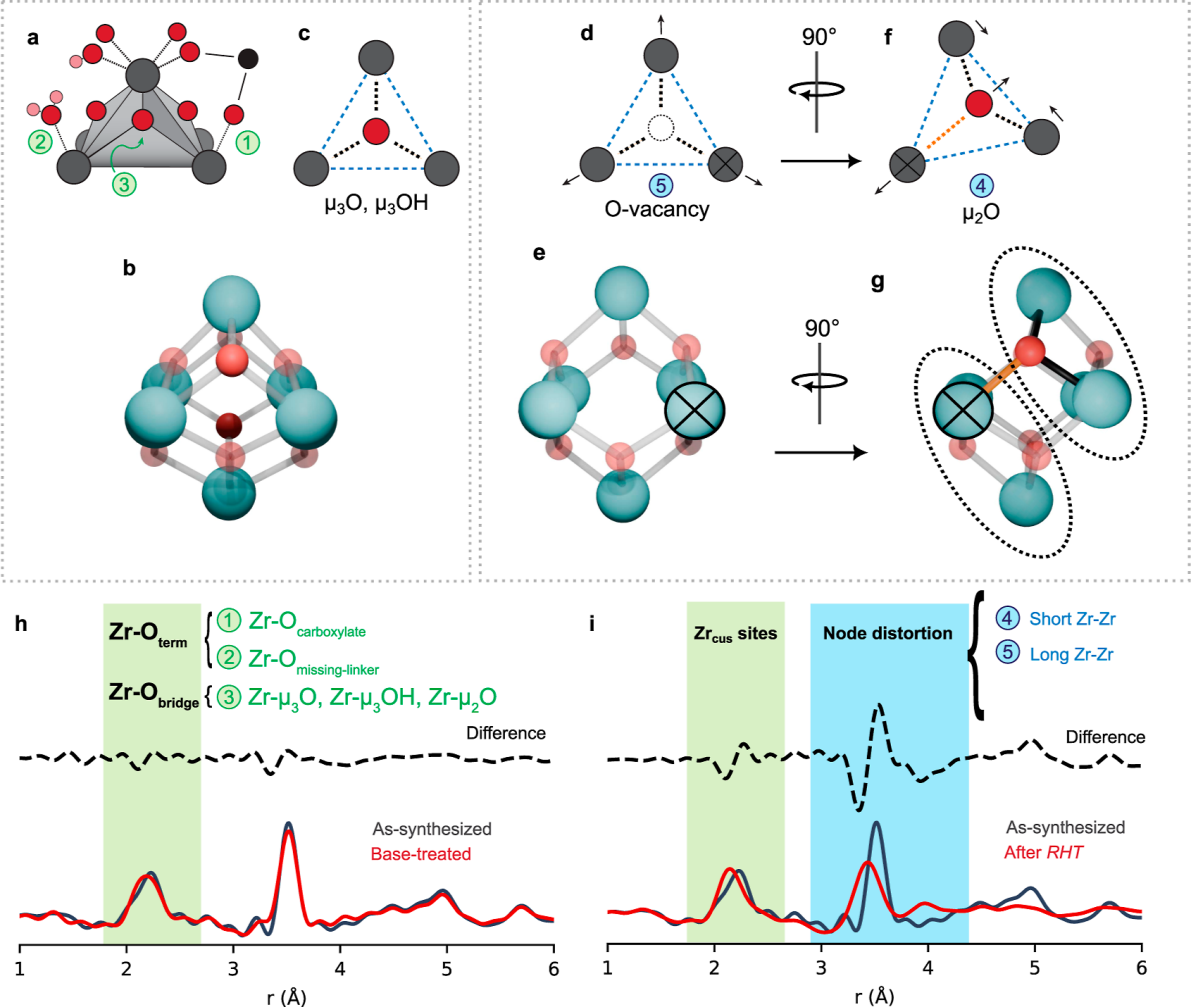
Structural transformations and corresponding signature changes in the PDF of defective {Zr_6_} nodes. The top-left (a-c) illustrates the hydrated cluster, and the top-right (d-g) illustrates the distorted dehydroxylated cluster. Panels (f) and (g) offer side-views, rotated by 90^◦^, of panels (d) and (e), respectively. The atoms with the black cross indicate the identity of a reference atom through the 90° rotation. (a) Classification of Zr–O bonds in the top half of the cluster. (b) The hydrated Zr_6_O_8_ cluster; dark red highlights the two bridging μ_3_OH groups removed during dehydroxylation. The remaining bridging oxygens are shown in transparent form. For clarity, the terminal O groups have been removed. (c) In the hydrated state, μ_3_O/μ_3_OH groups cap the octahedral faces. (d) Removing these groups creates O-vacancies, leading to elongated Zr–Zr pairs. (e) The resulting dehydroxylated cluster loses its octahedral symmetry. Consequently, (f) the remaining bridging oxygens transition to μ_2_O groups bridging two neighboring Zr atoms, which (g) shortens the respective Zr–Zr pairs. The dashed circles group Zr atoms with shortened Zr–Zr pair distances. (h) Missing-linker defects cause minor broadening of the Zr–O peak and slight distortions of the Zr–Zr distances in the PDF. In contrast, (i) cluster dehydroxylation significantly shifts the Zr–O peak to lower-*r* and causes pronounced splitting of the Zr–Zr distances.

We note that previous literature indicates that cluster dehydroxylation at low temperatures is reversible upon re-exposure to atmospheric moisture.^24^ However, it has been shown that at higher temperatures, node distortions can get kinetically trapped^41^ and that the presence of linker defects creates irreversible node distortions.^20^ We believe that this kinetic trapping comes from a combined effect of {Zr_6_} dehydroxylation and missing-linker defects. Specifically, we have demonstrated that irreversible node distortions occur in DUT-67 during *RHT* at 300 °C (573 K); while the elevated temperature drives cluster dehydroxylation, the humidity in the gas stream simultaneously hydrolyzes metal—ligand bonds, creating terminal OH^−^/H_2_O defect sites that we believe stabilize the distorted node geometry.^22^

To quantitatively track the cluster transformation, we developed representative structural models for the nondistorted and the distorted nodes. For this, we used the DFT-optimized hydrated Zr_6_O_8_ and dehydroxylated Zr_6_O_6_ clusters from our previous work.^22^ Note that only Zr and O atoms were included in the refinement, since C and H contribute little to the PDFs. Further, for simplicity, we denote the two clusters as Zr_6_O_8_ and Zr_6_O_6_ without writing out the full chemical formulas which include terminal ligands; Zr_6_O_8_ ≔ Zr_6_O_8_^bridging^O_24_^terminal^ and Zr_6_O_6_ ≔ Zr_6_O_6_^bridging^O_24_^terminal^, respectively. We first fitted the PDF of the parent MOF with the pristine Zr_6_O_8_ cluster using three refinement strategies: (a) keeping the cluster frozen in its DFT-optimized geometry, (b) allowing the cluster to expand or contract isotropically (a “breathing” model), and (c) allowing unconstrained atomic displacements of up to ±0.3 Å (Figure S6a–c). The frozen model yielded the poorest fit (Table S4), systematically overestimating the Zr–Zr distances. While the breathing cluster significantly improved the fit of the short edge-sharing Zr–Zr distances around 3.5 Å through a slight contraction (see zoomscale parameter in Table S5), it simultaneously worsened the fit for the diagonal Zr–Zr pairs near 5 Å (Figure S6b). This indicates that the discrepancy is not merely a uniform mismatch in bond lengths, but rather that the high symmetry of the DFT model fundamentally fails to capture the true distribution of Zr–Zr distances in the material. To understand the nature of this structural mismatch, we analyzed the atom-position relaxations from the unconstrained fits for both the pristine Zr_6_O_8_ cluster against the parent MOF (Figure S6c, Table S6) and the dehydroxylated Zr_6_O_6_ cluster against UiO-66-RHT-40 (Figure S6d, Table S7). The DFT-optimized and subsequently refined structures are depicted in Figures S8 and S7.

To understand how much the two {Zr_6_} structures changed after refinement, the mean Zr and O atom-displacements were calculated using eq ([Disp-formula eq4]). Comparing the magnitude of displacements in both refinements (Table 1), we find that the mean displacements of Zr and O atoms were almost the same in both cases. This was surprising, because we assumed the DFT-optimized pristine cluster (Zr_6_O_8_) would fit the experimental PDF of the parent UiO-66 better than the dehydroxylated cluster would fit the PDF of UiO-66-RHT-40.

**Table 1:** Magnitude of Displacement of Each Atom During the Refinement was Calculated Using eq 4

cluster	mean atom-displacement (Å)^a^
Zr_6_O_8_	All = 0.25 ± 0.07
	Zr = 0.19 ± 0.06
	O = 0.26 ± 0.06
Zr_6_O_6_	All = 0.26 ± 0.07
	Zr = 0.19 ± 0.09
	O = 0.27 ± 0.05

a The mean displacement average over all atoms, and per element, was calculated and is shown here. The errors are standard deviations. The displacements are calculated between the DFT-optimized starting model and the final refined model after fitting the Zr_6_O_8_ to the as-synthesized parent MOF and the Zr_6_O_6_ to UiO-66-RHT-40.

To better understand this, we compare the DFT-optimized and the refined models. We briefly summarize the findings here, but more detail is available in the Supporting Information (Supporting note S5). We first discuss how the original DFT-optimized dehydroxylated cluster was modified to fit the PDF of UiO-66-RHT-40, and then how the DFT-optimized hydrated cluster was modified to fit the PDF of the parent MOF. In short, the dehydroxylated DFT-optimized starting model did not reproduce the peak splitting of the edge-sharing Zr–Zr distances exactly. The refinement adjusted the model by moving the nonbridged Zr atoms further away from each other (Figure S7 and S8 and Table S8 and S9). On the other hand, the mean Zr–O distances, grouped into bridging and terminal groups as described in Figure 2b, change only minimally during the refinement.

Interestingly, refining the pristine DFT-optimized model against the data of the parent MOF revealed a similar trend: edge-sharing Zr–Zr pairs split into short (3.32 Å) and long (3.75 Å) distances to fit the second-shell peak of the PDF (Figure S8 and Table S9). While previous single-crystal XRD studies revealed substantial disorder in the oxygen groups capping the cluster (due to linker defects),^28,29^ our results indicate the Zr atoms themselves are also disordered in parent UiO-66 (powder form). We believe that such disorder is more likely to occur in polycrystalline powders compared to single crystals. Previous DFT calculations of ligand-capped {Zr_6_} cluster have shown that removing linking ligands or substituting them with terminal OH^−^/H_2_O groups leads to small distortions that create tiny peak splittings in the short and long Zr–Zr distances.^20,22^ We note that these distortions are, however, much less severe than what is observed after removing bridging μ_3_OH groups from the cluster.^22^ Thus, we believe that dehydroxylated clusters likely already exist in the as-synthesized powder. To further support this, the synchrotron PXRD pattern of the parent UiO-66 was assessed, which was found to have a large amorphous fraction of ∼ 40% (Figures S3). We note that previous EXAFS analysis has indicated that dehydroxylated clusters are present in as-synthesized powders of amorphous UiO-66.^23^

While literature often attributes the properties of Zr-MOFs to missing-linker defects, our data suggest that distorted nodes are inherently present in the as-synthesized powders. So, understanding the role of these distortions in structure–property relations is crucial. In the following sections, we first correlate the concentration of distorted nodes with the As­(V) adsorption capacity of UiO-66. We then systematically vary the number of missing-linker defects in amorphous UiO-66, keeping the amount of node distortions constant, in an effort to study the impact of each defect type on As­(V) uptake. We note that we cannot completely decouple the two, since the irreversible node distortions only occur in the presence of linker defects.^20^ However, the amount of linker defects can be varied.

### The Effect of Node Distortions

We conducted a two-phase model fit to each PDF (varying *RHT* time), using the DFT-optimized Zr_6_O_8_ (allowing isotropic expansion/contraction) and the refined dehydroxylated Zr_6_O_6_ cluster. We chose the refined model for the dehydroxylated cluster, instead of the DFT-optimized one, as the former allows us to better reconstruct the experimental PDFs with two phases. We note that modeling this highly disordered system with only two discrete clusters is an approximation. The real amorphous and semicrystalline intermediates exhibit a continuous landscape of local distortions driven by varying surface chemistries, such as the substitution of linking ligands with terminal OH^−^/H_2_O groups, and by varying degrees of cluster dehydration. Because our small-box approach absorbs this broad structural heterogeneity into just two representative phases, the fits naturally yield some unideal atomic displacement parameters (ADPs) (Table S10) and likely slightly overestimate the fraction of the dehydroxylated phase–particularly for the parent MOF and early *RHT* stages. Nevertheless, this two-phase fitting successfully captures the average structural evolution, providing a robust, representative metric for the degree of node distortion as a function of *RHT* time. A representative selection of fits is shown in (Figure 3a), and the refinement for all samples (*RHT* 1−40 min) is shown in Figure S9 and Table S10.

**Figure 3:**
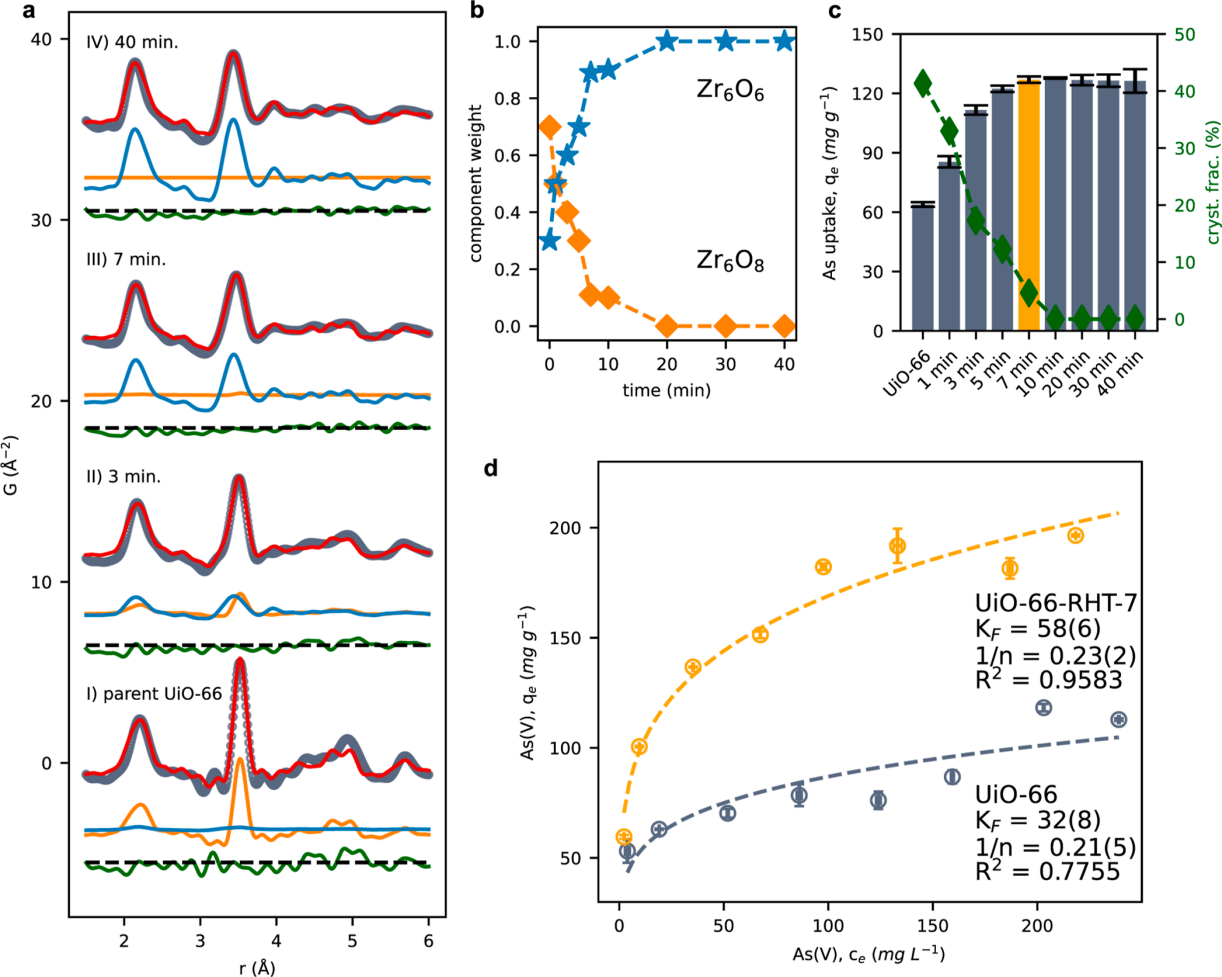
(a) Two-phase model fits of the local structure in UiO-66 are shown for the as-synthesized, 3 min, 7 min, and 40 min treatment times. Phase 1 uses the DFT-optimized Zr_6_O_8_ cluster, representing pristine, defect-free clusters (light blue). Phase 2 uses the DFT-optimized dehydroxylated cluster, which was further refined by allowing the Zr and O atoms to move freely to fit the 40 min sample (orange). The linear combination of the two phases is plotted in red. Below each fit, the difference curve between the linear combination and the experimental data is plotted in green. Dotted black lines are guides to the eye. (b) Component weights (mole fractions) of each phase in the two-phase model fit as a function of *RHT* time. (c) As­(V) uptake was measured for each sample, together with the crystalline fraction percentage calculated from the SPXRD. Conditions of the As adsorption experiment: adsorbent dosage = 0.25 mg mL^−1^, *c*_0_ = 40 mg L^−1^, time = 48 h. (d) As­(V) isotherms measured for the parent UiO-66 and UiO-66-RHT-7. Experimental conditions: adsorbent dosage = 0.25 mg mL^−1^, time = 48 h. Error bars (in c and d) are standard deviations from triplicates.

Using this method, the PDF of the parent UiO-66 was fitted and found to have a component weight (mole fraction) of 30 ± 20% of the dehydroxylated {Zr_6_} nodes. After *RHT*, the mole fraction of the dehydroxylated cluster increases gradually until it reaches 100% after 20 min of treatment (Figure 3b). While prolonging the *RHT* beyond 20 min does not significantly change the local structure observed via PDFs or BET surface areas (Figure S2 and Table S3), TGA indicates that prolonged *RHT* continues to create missing-linker defects (Figure S1 and Table S1).

To assess how the structural defects affect As­(V) uptake by UiO-66, batch adsorption experiments were conducted over a period of 48 h. The As­(V) uptake increases gradually until it plateaus after 7 min of *RHT* (Figure 3c). After 7 min, UiO-66 is fully amorphous (Figure 1a), and according to TGA and PDF analysis, it consists mostly of dehydroxylated clusters (89 ± 9%, Table S10). Figures 3b,c suggests a positive correlation between the amount of distorted nodes and the As­(V) uptake capacities. However, because the density of linker defects also gradually increases during the *RHT*, we cannot definitively link the improved performance to the degree of node distortion. We will further investigate this in the section The Effect of Linker Defects. In contrast, there is a negative correlation between the crystalline fractions and the As­(V) uptake capacities, while BET surface areas seem to have little effect on the adsorption capacity.

To make a more conclusive comparison, As­(V) isotherms were measured for the parent UiO-66 and the best performing sample—amorphous material after 7 min of *RHT*, denoted as UiO-66-RHT-7—revealing that the latter outperforms the crystalline phase across a large concentration range (Figure 3d). The maximum As­(V) adsorption capacity of UiO-66-RHT-7 is roughly double that of the parent UiO-66 (∼200 mg g^−1^ compared to ∼ 100 mg g^−1^). Importantly, there is also a much steeper uptake in the low-concentration regime for UiO-66-RHT-7, indicating a large number of strong adsorption sites and a higher affinity of the material toward As­(V). This is also indicated by the fitted Freundlich parameters (Figure 3d); The larger values for *K* and *n* ^−1^ reflect that the isotherm is steeper and the material has energetically more favorable binding sites.^42^ TGA showed that UiO-66-RHT-7 has roughly 3.36% less organic linkers compared to UiO-66. This does, however, not account for the ∼100% increase in capacity, indicating that the {Zr_6_} in UiO-66-RHT-7 has a stronger affinity toward As compared to the high-symmetry cluster.

Next, kinetic As­(V) adsorption studies show that despite enhanced arsenic uptake capacities, the adsorption kinetics are slower (Figure S10 and Table S11), which is likely linked to the reduced porosity and pore collapse induced by *RHT*. This observation suggests that the improved As­(V) uptake capacity is due to local node distortions, rather than amorphization. In principle, the amorphous state could offer beneficial properties for water purification, such as a broader, more hierarchical pore-size distribution than crystalline analogs, thereby reducing mass-transport limitations. However, in the specific case of UiO-66-RHT-40, mass transport appears to be more hindered than in the crystalline parent MOF. Therefore, the increase in total uptake capacity must be driven by the altered local structure—specifically, the creation of highly reactive Zr_cus_ sites that provide stronger and more abundant binding sites for As­(V).

This data suggests that engineering the local structure at the expense of long-range order can yield beneficial properties in Zr-based MOFs. Further, the results indicate that node distortions and cluster dehydroxylation may be an effective means of defect engineering in Zr-MOFs to tune their properties. The partial collapse of pores due to amorphization has a negative impact on the adsorption kinetics, but we believe that this could be dealt with by using template^43–45^ or template-free^46^ strategies to induce meso- and macropores in the amorphous materials. In the context of As­(V) removal, it seems that cluster dehydroxylation creates new adsorption sites for oxyanions. Below, we discuss possible binding modes and how different defect types may influence the As­(V) removal efficiency.

### As­(V) Binding Modes

Figure 4a shows possible hypothetical binding modes between As­(V) and {Zr_6_} nodes, highlighting the role of defects and vacancies around Zr atoms to facilitate the formation of new bonds to As­(V) species. While previous literature has confirmed As­(V) binding to typical hydrated {Zr_6_} nodes via EXAFS,^38,47^ its interaction with distorted, dehydroxylated clusters remains unexplored. To investigate this, we used differential PDF (dPDF) analysis on UiO-66-RHT-40 and parent UiO-66 before and after As­(V) adsorption Figure 4b,c. Notably, the dPDF of the As-loaded UiO-66-RHT-40 exhibits highly structured features even at low concentrations (120 mg L^−1^) (Figure 4b), whereas that of the As-loaded parent MOF contains mostly noise even at high As­(V) loadings (250 mg L^−1^) (Figure 4c). This indicates more well-defined As­(V) binding modes on the dehydroxylated clusters compared to the hydrated nodes.

**Figure 4:**
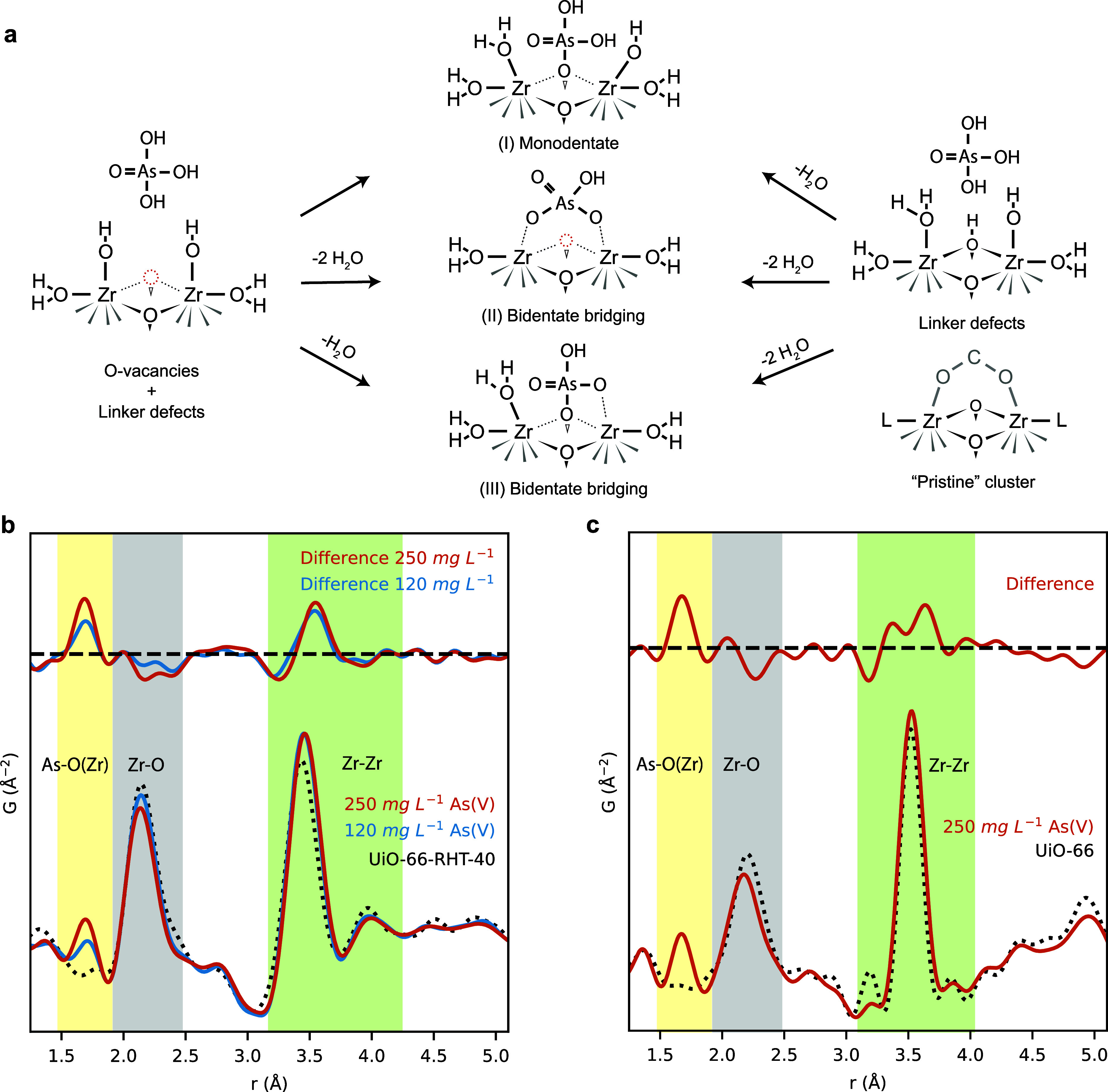
(a) Schematic representation of the surfaces of {Zr_6_} clusters with different surface defects and possible binding modes with As­(V). Dotted red lines denote O-vacancies (missing μ_3_O/μ_3_OH groups). For simplicity, we denote one of the bridging μ_3_O groups as vacant in binding mode (II), but for the nondehydroxylated starting cluster (right side), this site would obviously not be vacant. (b,c) Comparison of PDFs of arsenic-loaded samples with their respective unloaded counterparts for the fully dehydroxylated cluster material UiO-66-RHT-40 (b) and the parent UiO-66 (c). PDFs before and after arsenic loading were scaled using PDFmorph.^49^ The differential curve was then obtained by subtracting the scaled PDFs.

Further, the signals in the dPDF reveal three key structural changes upon As­(V) binding. First, a new peak in the dPDFs at 1.7 Å emerges, associated with As–O bonds in HAsO_4_^2−^/H_2_AsO_4_^−^. Second, negative peaks between 2.1 and 2.2 Å, a result of more narrow Zr–O signals in the As-loaded samples, suggest the displacement of terminal OH^−^/H_2_O groups, as suggested by others.^48^ Lastly, additional peaks appear around 3−4 Å. These likely correspond to new Zr–As pairs, matching previous EXAFS studies in As-loaded UiO-66,^38,47^ and new Zr–Zr distortions caused by monodentate or bidentate bridging (Figure 4a).

Since *RHT* also induces a small number of missing-linker defects alongside cluster dehydroxylation, it is necessary to assess how each influences As­(V) uptake in UiO-66-RHT-40. Below, we explore how the density of linker defects in UiO-66 during and after *RHT* can be enhanced and how this affects the As­(V) uptake of the MOFs.

### The Effect of Linker Defects

While we have observed a positive correlation between the number of dehydroxylated {Zr_6_} nodes and As­(V) uptake, the concurrent formation of missing-linker defects during *RHT* must also be considered. Based on this and previous work,^22^ avoiding linker-defect formation during *RHT* is likely not possible—and may in fact be undesirable, as we believe these defects play a role in stabilizing the node distortions. Furthermore, the ligand decomposition temperatures generally exceed the temperature at which cluster dehydroxylation occurs, making independent control of these two processes challenging. Nevertheless, it may be possible to target missing-linker defects independently—either by promoting greater linker burnoff during *RHT* or by introducing missing-linker defects independently through chemical treatment. To this end, we explore two strategies: (i) modifying UiO-66 to lower the ligand “burnoff” temperature during *RHT*, and (ii) applying chemical treatments pre- and post-*RHT*. Unlike thermal methods, chemical treatments are expected to induce missing-linker defects in the parent MOF without triggering cluster dehydroxylation.^36^ These approaches are summarized in Figure 5.

**Figure 5:**
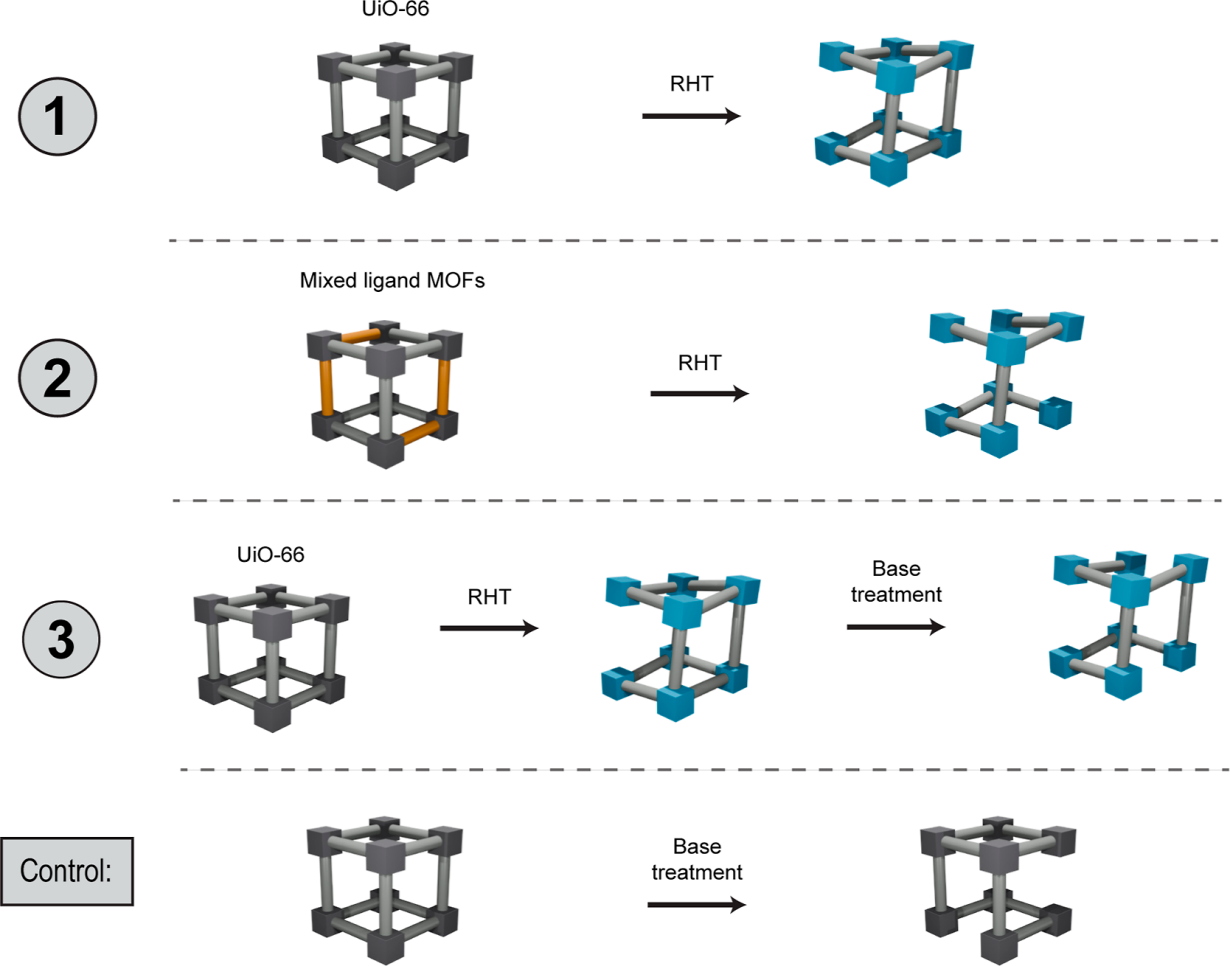
Schematic overview of the three chosen approaches to introduce missing linkers. Cubes represent the inorganic {Zr_6_} nodes, and the cylinders represent organic linkers. The change in color for the cubes from gray to blue indicates that the *RHT* changes the chemical and physical properties of those clusters (node distortions). Cylinders with different colors (gray and yellow) represent different organic ligands.

Method 1 serves as the baseline, applying a 7 min *RHT* to the parent UiO-66. This duration was chosen because As­(V) uptake plateaus at this point (Figure 3c) while maintaining minimal linker defects (1.40 out of 6, Table 2). Methods 2 and 3 were designed to systematically increase missing-linker defects while matching the cluster dehydroxylation levels of Method 1. In Method 2, we incorporated thermolabile linkers into UiO-66. Since these decompose at lower temperatures than BDC^2−^, we expected them to yield an amorphous structure with higher defect concentrations dictated by the initial linker ratio. Alternatively, Method 3 uses a sequential approach: parent UiO-66 is first treated via *RHT* (as in Method 1), followed by a NaOH base treatment to hydrolyze metal−ligand bonds^36^ and further increase the missing-linker defect density. As a control, we also applied the base treatment to the parent UiO-66, thereby inducing more missing-linker defects without amorphatizing the material or creating dehydroxylated clusters. For all samples, we determined the missing-linker fractions using TGA and determined BET surface areas from nitrogen adsorption isotherms. Further, the local structure of each MOF after the treatments was compared to the baseline sample (Method 1), by applying the two-phase fitting used before (Tables S16–S19). The results are summarized in Table 2.

**Table 2:** Overview of Missing-Linker Fractions and Degree of Cluster Dehydroxylation for Each MOF After Treatment Protocols Described in Figure 5

method^a^	BDC-NH_2_ fraction	missing-linkers	cluster dehydroxylation	BET surface are	As(V) uptake	norm. As(V) uptake^c^
	(%)	(out of 6)	(%)	(*m* ^2^ *g* ^−1^)	(mg g^−1^)	(mg g^−1^)
method 1		1.40	89 ± 9	435	110.3 ± 2.4	≈333.53
method 2	14	1.64	90 ± 10	436	128.4 ± 0.4	≈374.78
	17	1.73	94 ± 7	407	122.6 ± 3.2	≈358.89
	20	1.82	94 ± 6	397	115 ± 2.4	≈323.21
	23	1.70	94 ± 7	390	109.6 ± 1.0	≈319.90
	25	1.93	97 ± 7	389	109.8 ± 0.9	≈312.99
	32	1.92	98 ± 6	24	102.9 ± 1.7	≈291.99
	38	2.19	100 ± 4	25	75.0 ± 3.1	≈204.02
	56	^b^	^b^	37	17.1 ± 0.2	≈26.98
	78	^b^	^b^	17	17.5 ± 0.1	≈29.53
	100	^b^	^b^	29	10.1 ± 0.5	≈15.56
method 3		2.65	90 ± 10	328	100.1 ± 3.9	≈312.71
control		2.43	50 ± 10	1107	57.1 ± 0.3	≈170.65
parent UiO-66		1.24	30 ± 20	1422	65.7 ± 0.2	≈243.06

a The percentage of dehydroxylated clusters was determined by fitting two phases (hydrated and dehydrated clusters) to the experimental PDF, as described in Section Effect of Node Distortions. The fitting results are summarized in Tables S16–S19. BET surface areas were calculated using BETSI,^50^ and the As­(V) uptake was determined from a batch adsorption experiment with a soaking time of 48 h, initial As­(V) concentration of 40 mg L^−1^, and an adsorbent dosage of 0.25 mg mL^−1^.

b Not calculated because PDF data shows formation of ZrO_2_.

c Measured As­(V) uptake divided by Zr wt % as determined from TGA (Table S2,S15 and S18).

First, we discuss the results from Method 2, where we used a previously reported system of mixed BDC and thermolabile BDC-NH_2_ linkers.^38^ Mixed-linker MOFs (ml-MOFs) could be obtained in high crystallinity (Figure S14a) with BDC-NH_2_ content ranging from ∼ 14−78% as determined by TGA (Figures S11–S13). During the *RHT* (7 min), all BDC-NH_2_ linkers were removed from the mixed linker MOFs (ml-MOFs), yielding amorphous structures composed exclusively of BDC linkers, as determined by NMR (Figure S18). Further, the amount of missing-linker defects after the *RHT* strongly correlates with the BDC-NH_2_ fraction in the parent MOFs (Table 2). With Method 2, we could create amorphous MOFs with up to 2.19 missing linkers out of 6, determined by TGA (Table S14 and Figures S15–S17). Nitrogen adsorption isotherm measurements reveal that most ml-MOFs remained porous after *RHT*, except for those containing exceptionally large (>32%) fractions of BDC-NH_2_ (Table 2). Further, PDF analysis showed that ml-MOFs containing BDC-NH_2_ higher than 50% (55.5, 77.7, and 100%) were thermally too unstable and formed zirconia during *RHT* (Figure 6b). The rest formed amorphous MOFs with the signature distorted local structure associated with dehydroxylated clusters. Next, we investigated the As­(V) adsorption performance of ml-MOFs before and after *RHT*, including UiO-66 as a reference (Figure 6a). The labels on the *x*-axis are the respective fractions of BDC-NH_2_ in the ml-MOFs obtained as synthesized. The absolute numbers of missing linkers after the *RHT* are denoted with purple stars. Compared to the baseline (Method 1), amorphous MOFs with higher missing-linker fractions showed only a marginal improvement in As­(V) uptake capacity (Figure 6a). There seems to be an optimum around 1.64 missing linkers out of 6 (Figure 6a) with an As­(V) capacity of 128 mg g^−1^ compared to 110 of the baseline with 1.4 missing linkers. Increasing the missing-linker fractions beyond 1.7 worsens the performance slightly, and there is a sharp drop in performance for the samples that formed bulk zirconia during the *RHT* (Figure 6a,b). Overall, the results from Method 2 suggest that increasing the number of missing linker defects does not significantly affect As­(V) uptake capacity.

**Figure 6:**
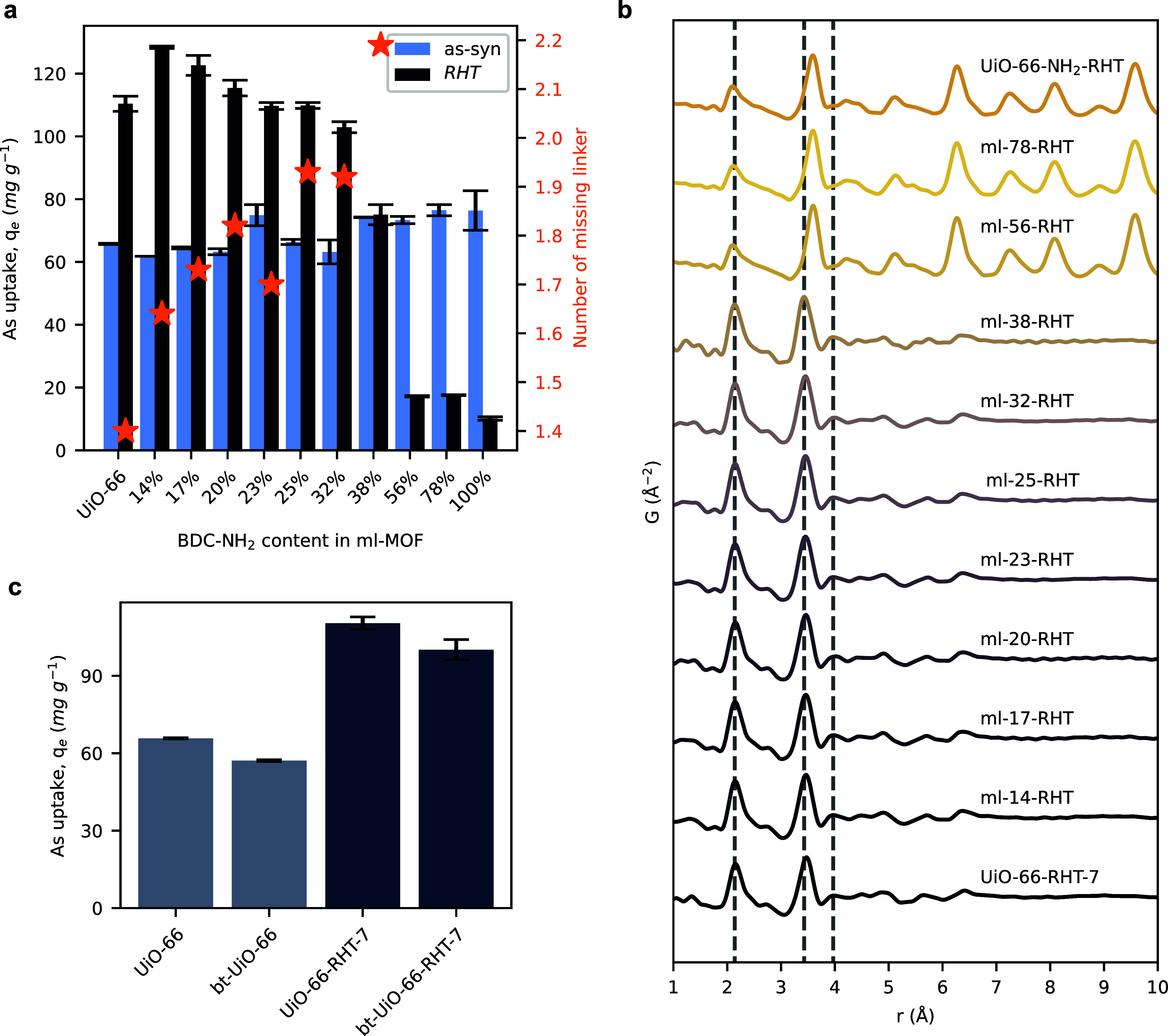
(a) Arsenic screening comparing mixed linker MOF, UiO-66, and UiO-66-NH_2_ with rapid heat-treated (7 min) mixed linker MOF, UiO-66, and UiO-66-NH_2_. The stars indicate the number of missing linkers obtained after the *RHT*. (b) PDFs of *RHT* mixed linker MOFs including *RHT* of UiO-66 (UiO-66-RHT-7) and pure BDC-NH_2_. The dashed lines are guides to the eye and are positioned at 2.14, 3.43, and 3.97 Å. (c) Arsenic batch adsorption experiment comparing UiO-66 and UiO-66-RHT-7 with base-treated UiO-66 and UiO-66-RHT-7, respectively.

To further verify that missing linkers do not enhance arsenic uptake, we applied a chemical treatment, known to induce such defects,^36^ to UiO-66-RHT-7 (Method 3) and to the parent UiO-66 (Control). The base-treated UiO-66 (bt-UiO-66) and the base-treated UiO-66-RHT-7 (bt-UiO-RHT-7) both remain porous (Figure S19) and have a significantly lower fraction of organic linkers (Figure S20a,b) compared to the parent UiO-66 and UiO-66-RHT, respectively. The number of missing linkers was calculated to be 2.43 and 2.65 for bt-UiO-66 and bt-UiO-66-RHT-7, respectively (Figure S20, Table S17), compared to only 1.24 for Method 1 (UiO-66-RHT-7, *RHT* only). Despite the increased density of missing-linker defects, the As­(V) uptake decreased after the base treatment for both materials (Figure 6c), providing further evidence that missing linker defects do not improve the As­(V) adsorption significantly.

PDF refinement of bt-UiO-66 revealed a higher fraction of 50 ± 10% of the lower symmetry, dehydroxylated cluster than the parent MOF (30 ± 20%) (Figure S21, Table S19). As stated earlier, our 2-phase model is highly approximate; rather than reflecting a genuine dehydroxylation, we believe the model compensates for minor distortions caused by missing linkers by inflating the weight of the dehydroxylated cluster component. Importantly, the base treatment, unlike the *RHT*, does not improve the materials’ As­(V) uptake. Taken together, none of the approaches used to introduce missing-linker defects led to a meaningful improvement in arsenic adsorption. These results collectively point to distorted {Zr_6_} nodes and Zr_cus_ sites—rather than missing linkers—as the key structural feature governing the performance in UiO-66.

## Conclusion

Using rapid heat treatment (*RHT*) under humid flow, we tracked the structural evolution of metal nodes in a Zr-MOF, namely UiO-66, from high-symmetry [Zr_6_O_4_(OH)_4_]^12+^ to distorted [Zr_6_O_6_]^12+^ clusters using PDF analysis. Our data indicates that these low-symmetry, dehydrated {Zr_6_} nodes are the primary driver of enhanced As­(V) adsorption performance—not missing-linker defects, as has often been assumed. Critically, increasing the number of missing linkers, whether through chemical post-treatments or *RHT* of mixed-ligand frameworks, fails to significantly enhance As­(V) uptake, indicating that node distortion and the resulting reduction in Zr coordination number are likely the key structural features governing performance. Beyond the context of arsenic remediation, our discovery of distorted nodes in as-synthesized UiO-66—which also exhibits an amorphous fraction of ∼40%—suggests that low-symmetry {Zr_6_} clusters are likely an intrinsic and previously overlooked feature of Zr-based MOFs. While defects in these materials have historically been discussed almost exclusively in terms of missing linkers and missing clusters, our findings point to a more nuanced picture in which node geometry plays a central and independent role. We expect that node defect engineering, as demonstrated here, may also prove broadly relevant across many applications where Zr-MOFs have shown promise. Thus, we believe accounting for local structural disorder is essential for advancing structure–property-function understanding in this important class of materials.

## Methods

### Synthesis of Materials

#### Synthesis of UiO-66

Zr-BDC was synthesized according to a previously reported literature procedure.^39^ In short, ZrCl_4_ (2.41 g, 1 equiv) was dispersed in DMF (180 mL) in a 1L cap-screw vessel and 18 mL of HCl (37 wt %) was added. Terephthalic acid (2.22 g, 1.29 equiv) was dissolved in DMF (360 mL), sonicated and added to the mixture. The reaction mixture was sonicated for 15 min, followed by heating at 80 °C for 24 h. After the reaction mixture was allowed to cool to room temperature, the solid was separated by centrifugation (5 min, 5000 rpm), washed with DMF (3 × 40 mL), and solvent-exchanged with EtOH (3 × 40 mL, overnight). The final product was dried at RT under vacuum for 12 h, yielding Zr-BDC as a white crystalline solid.

#### Synthesis of UiO-66-NH_2_

UiO-66-NH_2_ was synthesized according to a previously reported literature procedure.^39^ In short, ZrCl_4_ (2.41 g, 1 equiv) was dispersed in DMF (180 mL) in a 1L cap-screw vessel, and 18 mL of HCl (37 wt %) was added. 2-aminoterephthalic acid (2.42 g, 1.29 equiv) was dissolved in DMF (360 mL), sonicated and added to the mixture. The reaction mixture was sonicated for 15 min, followed by heating at 80 °C for 24 h. After the reaction mixture was allowed to cool to room temperature, the solid was separated by centrifugation (5 min, 5000 rpm), washed with DMF (3 × 40 mL), and solvent-exchanged with EtOH (3 × 40 mL, overnight). The final product was dried at RT under vacuum for 12 h, yielding UiO-66-NH_2_ as a yellow crystalline solid.

#### Synthesis of ml-MOF (BDC/BDC-NH_2_)

ml-MOFs were synthesized by adapting a previously reported literature procedure.^39^ In short, ZrCl_4_ (2.41 g, 1 equiv) was dispersed in DMF (180 mL) in a 1 L cap-screw vessel, and 18 mL of HCl (37 wt %) was added. 2-aminoterephthalic acid and terephthalic acid (total, 1.29 equiv) were dissolved in DMF (360 mL) in ratios of 0.05:0.95, 0.1:0.9, 0.15:0.85, 0.2:0.8, 0.25:0.75, 0.3:0.7, 0.4:0.6, 0.6:0.4, and 0.8:0.2, sonicated, and added to the mixture. The reaction mixture was sonicated for 15 min, followed by heating at 80 °C for 24 h. After the reaction mixture was allowed to cool to room temperature, the solid was separated by centrifugation (5 min, 5000 rpm), washed with DMF (3 × 40 mL), and solvent-exchanged with EtOH (3 × 40 mL, overnight). The final product was dried at RT under vacuum for 12 h, yielding mL-MOFs as crystalline solids.

#### Rapid Heat Treatment

The thermal treatment under humid gas flow was conducted by placing 300 mg of the parent UiO-66 in a ceramic boat and placing it in a preheated tube furnace at 400 °C. The tube furnace was under an air flow that had a water bubbler connected upstream to the furnace. The powder was exposed to the humid thermal treatment for 1, 3, 5, 7, 10, 20, 30, and 40 min. We note that the furnace temperature was set to 450 °C to achieve a temperature of 400 °C in the center of the tube furnace under humid flow, where the samples are placed. The temperature and relative humidity (RH) were measured just at the entry point of the furnace after the water bubbler. At this point, the RH was ∼96% at a temperature of 60 °C. From this, we can calculate the vapor pressure and the absolute humidity at 400 °C. First, we calculate the partial pressure of water at 60 °C and RH = 96%

$$RH\left[\%\right]=\frac{p_{H_{2}O}}{p_{H_{2}O}^{sat}}\times 100$$ 1

The saturation pressure $p_{H_{2}O}^{sat}\left(60^{{}^{\circ}}C\right)\approx 19600$ Pa can be obtained from water vapor tables.^51^ This gives us the partial pressure of water at our inlet point $p_{H_{2}O}\left(60^{{}^{\circ}}C\right)=19100$ Pa. From this, the absolute humidity (AH) can be calculated as follows

2 $$AH\left[g/m^{3}\right]=\frac{p_{H_{2}O}M}{RT}$$

where M is the molar mass of water (18.015 g mol^−1^), R is the universal gas constant (8.314 J/molK), and T is the absolute temperature in Kelvin (K). This gives us an AH­(60 °C) = 124.23 g/m^3^ at the entry point and at higher temperature inside the furnace AH­(400 °C) = 61.48 g/m^3^.

#### Base Treatment

The base treatment was carried out by soaking 100 mg of the parent UiO-66 and UiO-66-RHT-7, respectively, for 12h in Milli-Q water (100 mL) adjusted to pH of 9 using NaOH.

### Characterization

Nitrogen adsorption measurements were done on a Belsorp Max-II instrument at 77 K. The samples were activated at 150 °C prior to the measurement. The specific surface area was calculated by the BET method using BETSI.^50^ Thermogravimetric analysis (TGA) profiles were obtained using a TA Q-Series TGA Q500. The balance flow rate was 15 mL min^−1^ with nitrogen. The thermal stability profile was measured using 15 mL min^−1^ airflow and a ramp of 5 °C per minute up to 800 °C.

### Crystalline Fraction Calculations

We calculated the crystalline fractions in each material from synchrotron powder X-ray diffraction (SPXRD) data. The fractions were calculated as follows

$$F_{cryst.}\left(\%\right)=\frac{I_{m}-I_{poly}}{I_{m}-I_{bkg}}\times 100$$ 3

Where *I*_*m*_ is the total integrated intensity of the measured sample, *I*_poly_ is the integrated intensity of the fitted Chebyshev polynomial (representing the diffuse amorphous scattering), and I_bkg_ is the integrated intensity of the fitted background signal from a Si standard measurement. All integrations were performed by summing the intensities across the full measured 2θ range. The Chebyshev polynomial is used to fit the shape of *I*_*m*_, but excluding sharp Bragg peaks. For an amorphous sample, we would have *I*_poly_ = *I*_*m*_. The calculated crystalline fractions are plotted in Figure 3. As examples, the plotted fitted signals for the as-synthesized UiO-66 and after *RHT* for 3 min are shown in Figures S3 and S4.

### Pair Distribution Function Analysis

Total scattering measurements were conducted at BM31/Swiss Norwegian Beamlines (SNBL) at the European Synchrotron Radiation Facility (ESRF) in Grenoble, France, during two beamtimes (i) A31−1−205 and (ii) A31−1−271. In general, powdered samples were packed in glass capillaries with an outer diameter of 1 mm and a wall thickness of 0.01 mm. The samples were mounted on a capillary spinner downstream of the beam. A PILATUS3 X CdTe 2 M detector was placed at an angle of 15° downstream of the sample to maximize the accessible Q-range. Slightly different geometries and X-ray energies were used at the two separate beamtimes. PXRD measurements were done with (i) a wavelength of 0.25448 Å and a sample-to-detector distance of 0.89115 m, and (ii) a wavelength of 0.24486 Å and a sample-to-detector distance of 0.88888 m. Total scattering measurements were performed with (i) a wavelength of 0.25995 Å and a sample-to-detector distance of 0.19115 m, and (ii) with a wavelength of 0.244865 Å and a detector distance of 0.18888 m. An empty glass capillary was measured and subtracted from the 1D diffractograms prior to data reduction. Ten frames with an exposure time of 42 s were collected, averaged, and integrated using the SNBL Software Bubble.^52^ Data reduction to *G­(r)* was done using PDFgetX3 ^53^ withing xPDFsuite.^54^ The Fourier transform from *F*(*Q*) to *G*(*r*) was done using *Q*_max_ = 24.2 Å, *Q*_min_ = 0.7 Å, and *rpoly* = 1.0. Modeling and fitting of the PDF data were done using DiffPy-CMI.^55^ Structural models were obtained via first-principle DFT calculations from previous literature.^22^ For our purpose, all C and H atoms were removed from the DFT-optimized models. More details can be found in the Supporting Information (Supporting note S5). The magnitude of displacement of atoms during structural refinements was calculated as follows

4 $$displacement=\sqrt{{\left(x_{0}-x_{1}\right)}^{2}+{\left(y_{0}-y_{1}\right)}^{2}+{\left(z_{0}-z_{1}\right)}^{2}}$$

Where *x*, *y*, and *z* refer to Cartesian coordinates in units of Å, and the subscripts refer to the atom of the same identity before (0) and after (1) structural refinement. The “before” structure is the original DFT-optimized model.^22^

### Arsenic Adsorption Experiment

In general, for arsenic adsorption experiments, a stock solution of 250 mg L^−1^ was prepared by dissolving As_2_O_5_ (95.75 mg) in Milli Q water (250 mL). After adjusting the pH to 7 using HCl and NaOH, the solution was diluted to appropriate concentrations for the arsenic adsorption experiment.

### Batch Adsorption Experiment

All batch adsorption experiments were measured in triplicates. For this, 5 mg of dry MOF powder was weighed out in a 40 mL glass vial, followed by adding 20 mL of arsenic solution. The vials were closed and kept shaking (200 rpm) at room temperature for 24 h. The samples were then filtered through 0.22 μm pore PTFE syringe filters into a 15 mL centrifuge tube. From this solution, 4 mL was transferred to another 15 mL centrifuge tube, and 0.085 mL nitric acid (69%) was added. The arsenic concentration was then quantified using ICP-OES. Adsorption capacities and arsenic removal were calculated based on the different arsenic concentrations before and after adding the MOF powder to the solution. (see eq 5 and eq 6).Calculation of equilibrium adsorption capacity (Q_*e*_)­

$$Q_{e}=\frac{(c_{0}-c_{e})\;V}{m}$$ 5

*Q*_*e*_ is the amount of arsenic adsorbed at equilibrium (mg g^−1^); *c*_*e*_ is the concentration of arsenic at equilibrium mg L^−1^; *c*_0_ is the initial concentration of arsenic (mg L^−1^); *V* is the volume (L) of the metal ion solution; *m* is the mass of adsorbent (g)­The percentage removal is calculated as follows

6 $$Removal\%=\frac{c_{0}-c_{e}}{c_{0}}\times 100$$

where *c*_0_ is the initial concentration of arsenic (mg L^−1^) and *c*_*e*_ is the concentration of arsenic at equilibrium (mg L^−1^).

### Arsenic Isotherm Measurements

For isotherm measurements, 5 mg of dry MOF powder was weighed out in a 40 mL glass vial, followed by adding 20 mL arsenic solution with different concentrations (20–300 mg L^−1^). Arsenic isotherm data were fitted using the Freundlich equation

$$Q_{e}=K\;c_{e}^{1/n}$$ 7

Where *Q*_*e*_ is the amount of arsenic adsorbed at equilibrium (mg g^−1^); *c*_*e*_ = the concentration of arsenic at equilibrium (mg L^−1^); *K* the Freundlich constant and $\frac{1}{n}$ a parameter often associated with the strength of binding. The Freundlich model is purely empirical and is not derived from a physical model. Hence, the interpretation of fitted parameters should be done with reservation.

### Arsenic Kinetic Experiments

For kinetic experiments, 62.5 mg of dry MOF powder was weighed out in a 500 mL glass jar, followed by adding 250 mL arsenic solution (50 mg L^−1^). Aliquots (2 mL) were taken at different time points, followed by analysis via ICP-OES. Arsenic kinetic data were fitted using the linearized pseudo-second order model (PSO)

8 $$\frac{t}{Q_{t}}=\frac{1}{k_{2}^{2}Q_{e}^{2}}+\frac{t}{Q_{e}}$$

Where *Q*_*e*_ is the amount of arsenic adsorbed at equilibrium (mg g^−1^); *k*_2_ is the rate constant for PSO model (*g* (mg min)^−1^); *t* is the time (min).

## Supplementary Material



## Data Availability

Experimental data presented in this work and molecular structure files are openly available on Zenodo at: 10.5281/zenodo.20415497.

## Notes

Supporting note S1: TGA of UiO-66-RHT series (Figure S1 and Tables S1–S2). Supporting note S2: N_2_ adsorption isotherms of UiO-66-RHT series (Figure S2 and Table S3). Supporting note S3: Crystal fraction calculations (Figures S3-Figures S4). Supporting note S4: Zr K-Edge XANES (Figure S5). Supporting note S5: Pair distribution function analysis (Figure S6-Figure S9 and Tables S4–S10). Supporting note S6: Arsenic adsorption kinetics (Figure S10 and Table S11). Supporting note S7: Characterization of mixed linker MOFs (Method 2) (Figures S11– S1 and Tables S12-Tables S15). Supporting note S8: Characterization of base treated MOFs (Method 3) (Figures S19– S21 and Tables S17–S19) (PDF)
