# Supplementary material for: Node Distortions as a Means of Defect Engineering in Zr-Based MOFs
Source: J Am Chem Soc. 2026 Jul 30;148(31):33229–42. doi: 10.1021/jacs.6c06764 (PMC13474933; doi:10.1021/jacs.6c06764)
Supplement: Supplementary file 1 [file ja6c06764_si_001.pdf]

Supporting Information for:

Node Distortions as a Means of Defect Engineering in Zr-based MOFs

Till Schertenleib,[†,‡,¶] Timo M. O. Felder,[†,‡] Nazanin Taheri,[‡] Wei Shi,[§,‡] Tanaporn Na Narong,[‖] Mehrdad Asgari,[⊥,#] Pattaraphon Rodlamul,[‡] Simon J. L. Billinge,[‖,@] and Wendy L. Queen[*,‡]

†*The authors have contributed equally to this work*

‡*Institute of Chemical Sciences and Engineering (ISIC), École Polytechnique Fédérale de Lausanne (EPFL), 1950 Sion, Switzerland*

¶*Department of Chemistry, University of Copenhagen, København Ø, 2100 Denmark*

§*Research Center for Analytical Sciences, Department of Chemistry, College of Sciences, Northeastern University, Shenyang 110819, China*

‖*Department of Applied Physics and Applied Mathematics, Columbia University, New York, NY 10027 United States*

⊥*Departement of Chemical Engineering and Biotechnology, University of Cambridge, CB3 0AS Cambridge, UK*

#*Lucy Cavendish College, University of Cambridge, Lady Margaret Rd, Cambridge CB3 0BU*

@*Materials Department, University of California, Santa Barbara, Santa Barbara, CA 93106, United States*

E-mail: wendy.queen@epfl.ch

Contents

S1 TGA of UiO-66-RHT series **3**

S2 N_2 adsorption isotherms of UiO-66-RHT series **6**

S3 Crystal fraction calculations **7**

S4 Zr K-Edge XANES **9**

S5 Pair distribution function analysis **10**

S1 Refinement of atom positions Zr clusters 12

S1.1 UiO after *RHT* 13

S1.2 Parent UiO-66 (as-synthesized) 15

S2 Two-phase fitting results 17

S6 Arsenic adsorption kinetics **19**

S7 Characterization of mixed linker MOFs (Method 2) **20**

S1 Linker fraction analysis in mixed linker MOF using TGA 20

S2 PXRD and N_2 adsorption isotherm 24

S3 TGA of mixed linker (BDC/BDC-NH_2) MOFs after *RHT* 25

S4 PDF analysis of ml-RHT series 28

S8 Characterization of base treated MOFs (Method 3) **29**

S1 TGA of base-treated UiO-66 (bt-UiO-66) and UiO-66-*RHT*-7 29

S2 PDF analysis of base treated MOFs 31

References **33**

S1 TGA of UiO-66-RHT series

Figure S1a and b show the TGA data of as-synth UiO-66 and samples exposed to the RHT for different time intervals. The samples are named according to their treatment time as UiO-66-RHT-X, where X is the number of minutes of *RHT*. The weight loss between 25-200 °C can mainly be attributed to the loss of solvent molecules and and physisorbed water. The weight-loss between 200-400 °C (highlighted in Figure S1b) is attributed to the dehydroxylation of the Zr-oxo cluster and the removal of monodentate ligands (OH^-, H_2O and modulators). We assume that in the dehydroxylated material the charge of missing linker is compensated by an oxide anion on the cluster which leads to the chemical formula $Zr_6O_6(O)_x(BDC)_{6-x}$ where x is the number of missing linkers. Next, we assume complete decomposition of organic linkers at 800 °C to H_2O and CO_2 forming ZrO_2. Therefore, 1 mole of MOF turns into 6 moles of ZrO_2. The theoretical weight loss can then be calculated using Equation (1) and Equation (2), respectively;

$$\mathrm{MW_{MOF}} = \frac{W_{400^\circ\mathrm{C}}}{W_{800^\circ\mathrm{C}}} \times 6M_{\mathrm{ZrO_2}}. \tag{1}$$

When we set $W_{800^\circ C}$to 100 % and rearrange to $W_{400^\circ C}$ we get

$$W_{400^\circ\mathrm{C}} = \frac{\mathrm{MW_{MOF}}}{6M_{\mathrm{ZrO_2}}} \times 100. \tag{2}$$

Using the above equation, a defect-free UiO-66 shows a theoretical weight of 220.21 wt.%. The parent UiO-66 shows an experimental weight of 195.35 wt.%, which leads to a structural formula $ZrO_6(O)_{1.24}(BDC)_{4.76}$. Consequently, the number of missing linkers in the parent UiO-66 is 1.24. Theoretical structural formulas and calculated amount of missing linkers of different *RHT* samples can be found in Table S1.

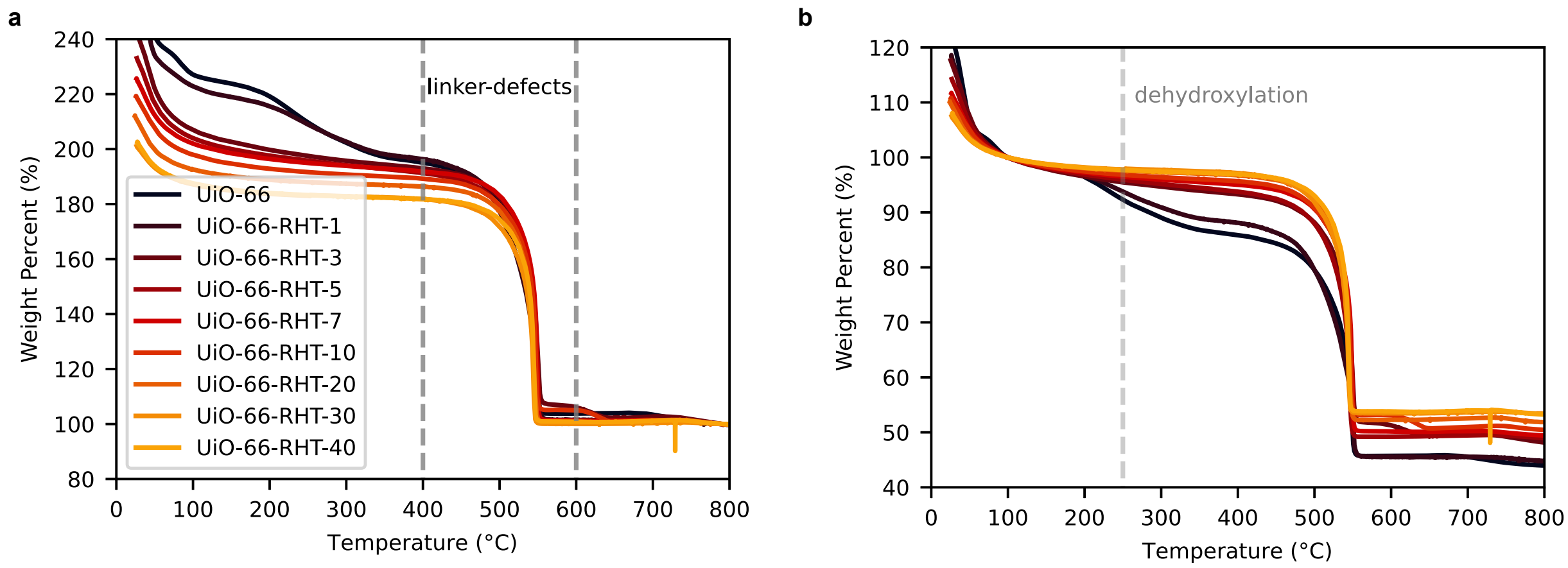

Figure S1: TGA profiles of UiO-$66_{\text{as-synth}}$ and samples exposed to the *RHT* for different time intervals. In (a), the TGA data are scaled by dividing each data set by the residual mass at 800 °C, so that the difference of the organic fraction in each sample is highlighted more clearly. In (b), each dataset is divided by the mass at 100 °C to highlight better the presence or absence of the cluster dehydroxylation step between 200-400 °C.

Table S1: Overview missing linker calculation results

material	wt. % norm to ZrO_2	Theoretical structure at 400 °C	missing linker	% and (absolute numbers) of missing linkers due to *RHT*
defect free UiO-66	220.21	$Zr_6O_6(C_8H_6O_4)_6$	0	-
Parent UiO-66	195.35	$Zr_6O_6(O)_{1.24}(C_8H_6O_4)_{4.76}$	1.24	0
UiO-66-RHT-1	196.61	$Zr_6O_6(O)_{1.18}(C_8H_6O_4)_{4.82}$	1.18	1.86 (-)
UiO-66-RHT-3	193.58	$Zr_6O_6(O)_{1.33}(C_8H_6O_4)_{4.67}$	1.33	1.89 (0.09)
UiO-66-RHT-5	191.46	$Zr_6O_6(O)_{1.44}(C_8H_6O_4)_{4.56}$	1.44	4.20 (0.2)
UiO-66-RHT-7	192.22	$Zr_6O_6(O)_{1.40}(C_8H_6O_4)_{4.60}$	1.40	3.36 (0.16)
UiO-66-RHT-10	189.59	$Zr_6O_6(O)_{1.53}(C_8H_6O_4)_{4.47}$	1.53	6.09 (0.29)
UiO-66-RHT-20	186.53	$Zr_6O_6(O)_{1.68}(C_8H_6O_4)_{4.32}$	1.68	9.24 (0.44)
UiO-66-RHT-30	181.85	$Zr_6O_6(O)_{1.91}(C_8H_6O_4)_{4.09}$	1.91	14.07 (0.67)
UiO-66-RHT-40	182.08	$Zr_6O_6(O)_{1.90}(C_8H_6O_4)_{4.10}$	1.90	13.8 (0.66)

To estimate the zirconium content in the samples, the TGA curves were normalized to the sample weight at room temperature. Assuming complete decomposition of the organic framework during heating and that the residual mass at 800 °C corresponds entirely to ZrO_2, the zirconium content can be calculated from the remaining mass fraction.

The mass fraction of Zr in ZrO_2 was calculated based on the molar masses of Zr and ZrO_2. Using this assumption, the Zr content in the samples was determined according to Equation (3), and are summarized in Table S2

$$\text{Zr wt.\%} = W_{800\,^{\circ}\mathrm{C}} \times \frac{M_{\mathrm{Zr}}}{M_{\mathrm{ZrO_2}}} \tag{3}$$

where $W_{800\,^{\circ}\mathrm{C}}$ is the residual mass fraction at 800 °C (normalized to the initial mass at room temperature), M_{Zr} is the molar mass of zirconium and M_{ZrO_2} is the molar mass of zirconium dioxide.

Table S2: Overview Zr content

**Material**	**ZrO_2 wt.%** at 800 °C	**Zr content in wt.%**
parent UiO-66	36.53	27.03
UiO-66-RHT-1	38.39	28.41
UiO-66-RHT-3	41.51	30.72
UiO-66-RHT-5	43.26	32.01
UiO-66-RHT-7	44.68	33.07
UiO-66-RHT-10	45.95	34.00
UiO-66-RHT-20	47.79	35.36
UiO-66-RHT-30	50.05	37.03
UiO-66-RHT-40	49.60	36.71

S2 N_2 adsorption isotherms of UiO-66-RHT series

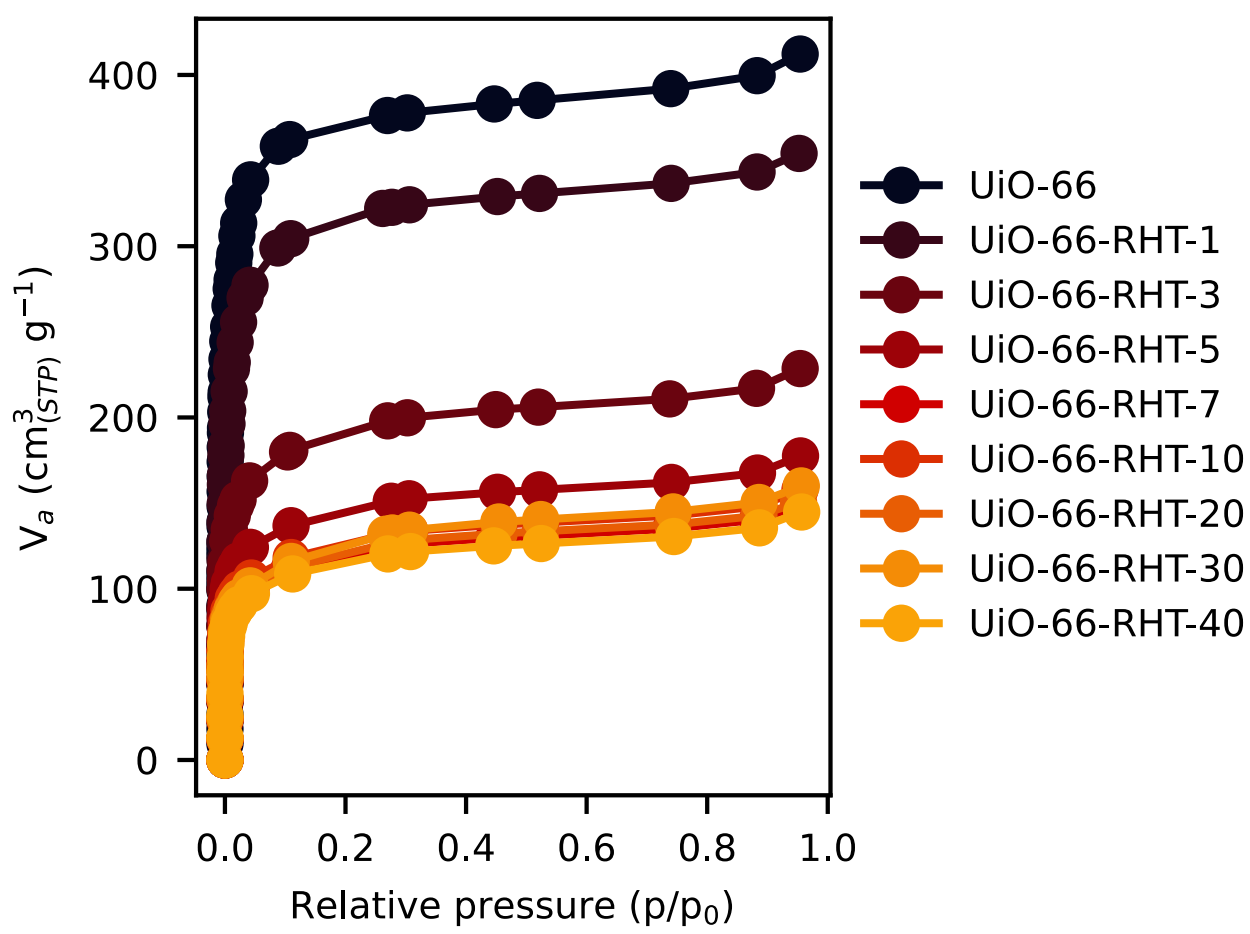

Figure S2: Nitrogen adsorption isotherm of the parent UiO-66 and samples exposed to the *RHT*.

Table S3: Brunauer–Emmett–Teller (BET) area of the parent UiO-66 and samples exposed to the *RHT*, obtained using BETSI[1]

Material	BET ($m^2\,g^{-1}$)
UiO-66	1422
UiO-66-RHT-1	1201
UiO-66-RHT-3	708
UiO-66-RHT-5	533
UiO-66-RHT-7	435
UiO-66-RHT-10	440
UiO-66-RHT-20	418
UiO-66-RHT-30	421
UiO-66-RHT-40	421

S3 Crystal fraction calculations

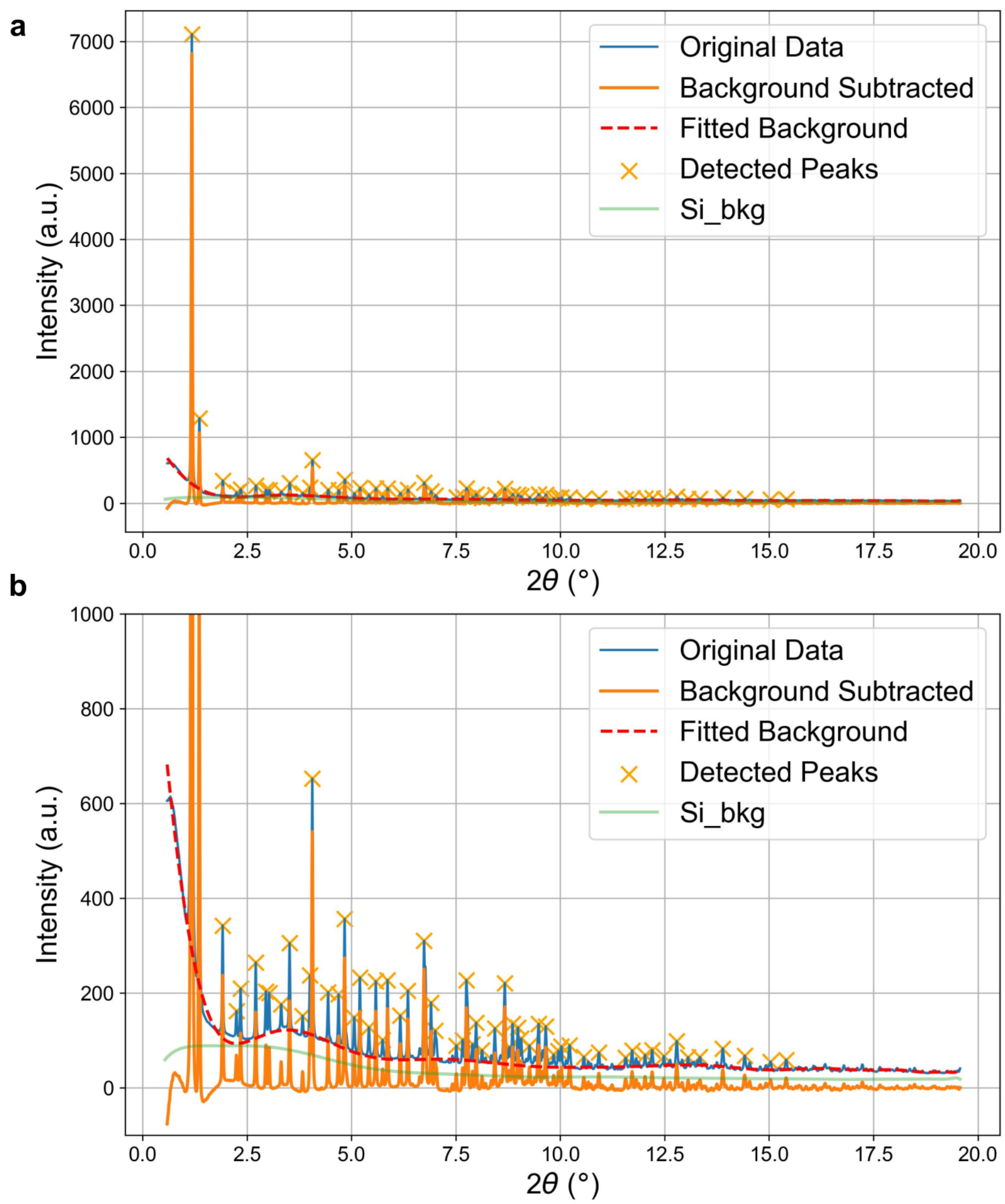

Figure S3: Determination of the crystalline and amorphous fraction in as-synthesized UiO-66 (a) and with an expanded y-scale in (b).

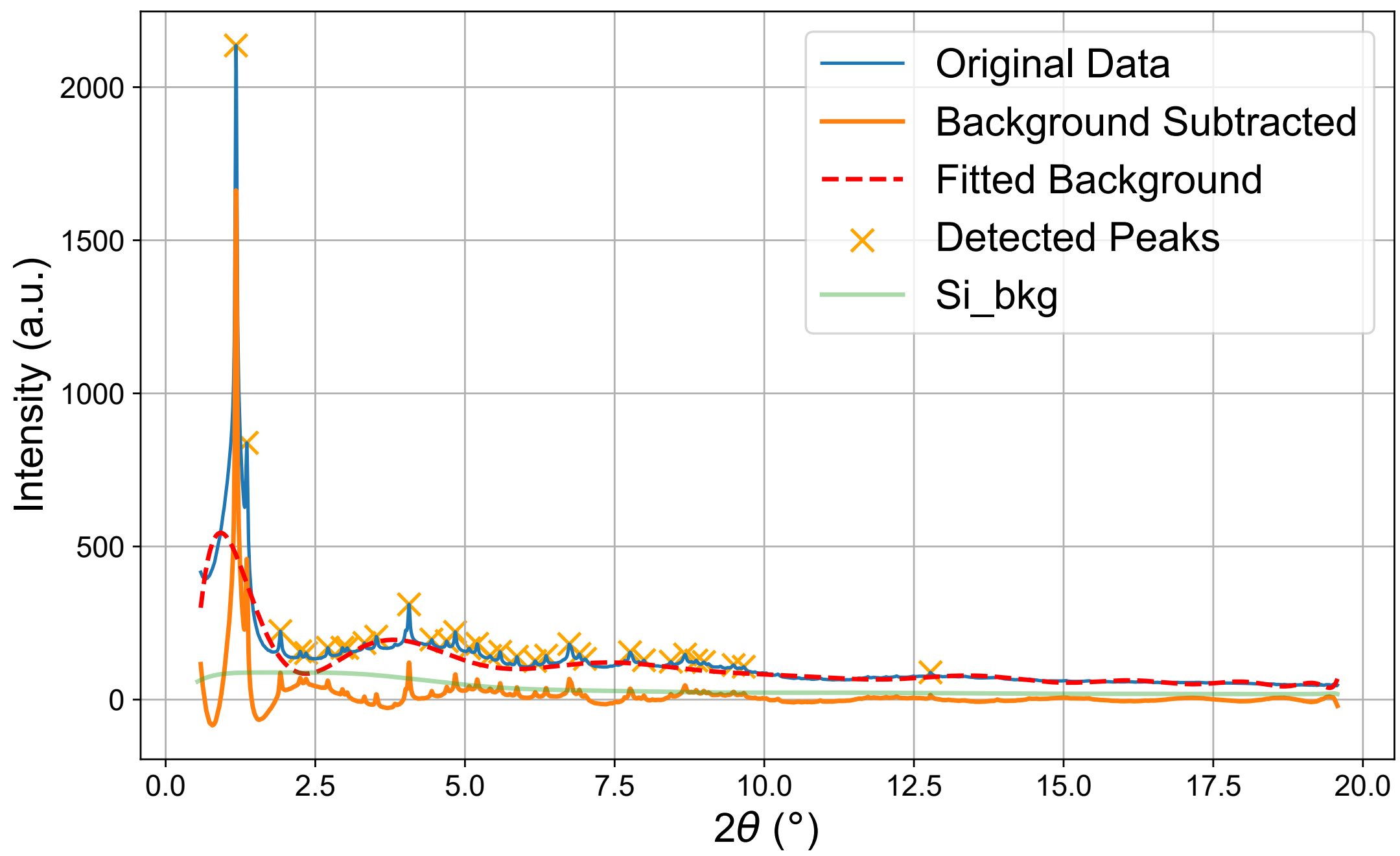

Figure S4: Determination of the crystalline and amorphous fraction in rapid heat treated UiO-66 (UiO-66-RHT-3).

S4 Zr K-Edge XANES

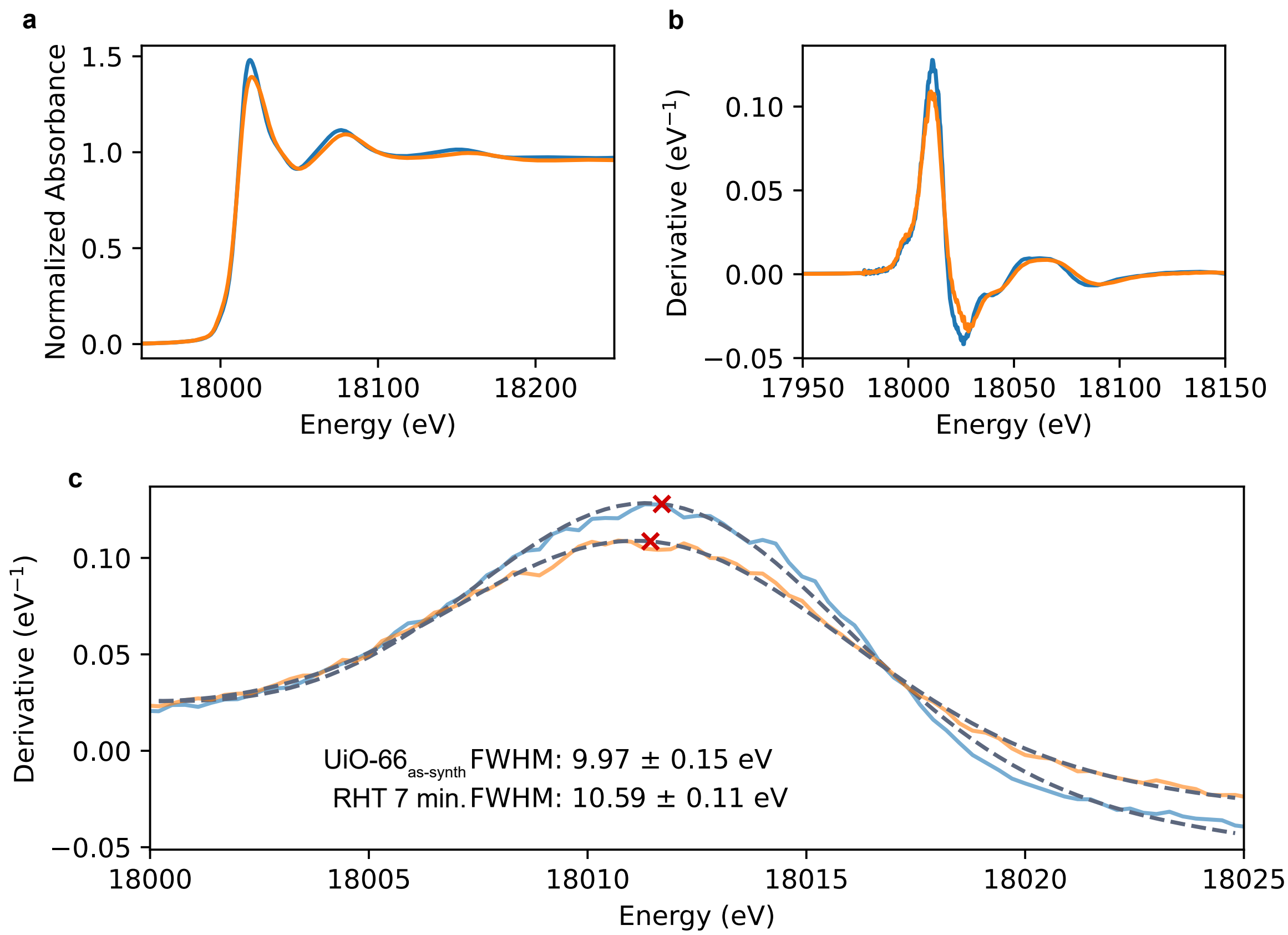

Figure S5: The data of the parent UiO-66 and UiO-66-RHT-7 are plotted in blue and orange, respectively. (a) Normalized XANES of the Zr K-edges. (b) The derivative of the XANES. (c) Gaussians were fitted (dashed lines) to the main peak in the derivative, and the full-width at half maximum (FWHM) is reported for both samples in the figure box, and the Gaussian centers are denoted as red crosses. The FWHM can be used as a diagnostic tool to compare the centro-symmetry of coordination environments around the absorber (Zr).[2]

S5 Pair distribution function analysis

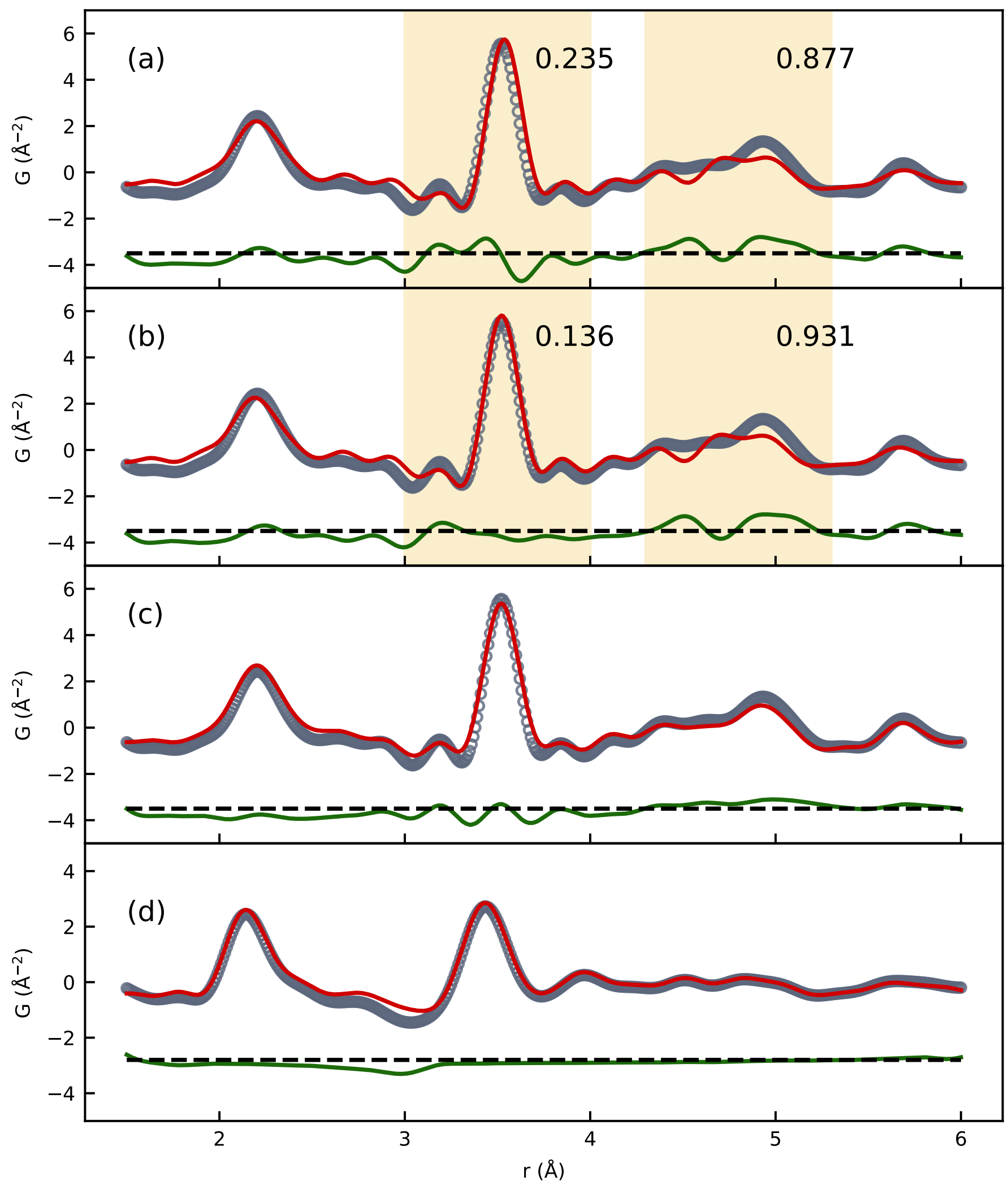

Figure S6: Single phase fit of the parent UiO-66 (a-c) UiO-66-RHT-40 (d). (a). The parent UiO-66 was fitted using the DFT-optimized {Zr_6} structural model with composition $Zr_6O_8{}^{Zr\text{-}Zr}O_{24}{}^{Zr\text{-}L}$ (Zr_6O_8). (b) The parent UiO-66 was fitted using the DFT-optimized Zr_6O_8 cluster as in (a), but the cluster was allowed to expand or contract isotropically to fit the data. The highlighted areas in (a) and (b) range from 3.0-4.0 and 4.3-5.3 Å. The numbers correspond to the calculated residual weight (R_w) calculated in the highlighted regions. The zoomscale parameter shrinks the Zr_6O_8 cluster in (b) to improve the fit of the second shell peak (3.0-4.0 Å) but worsens the fit of the long Zr-Zr pair (4.3-5.3 Å). (c) The parent UiO-66 PDF was fitted using the DFT-optimized Zr_6O_8 cluster, but the atoms were allowed to move in any direction by 0.3 Å. (d) UiO-66-RHT-40 was fitted using the DFT-optimized dehydrated structural model with composition $Zr_6O_6{}^{Zr\text{-}Zr}O_{24}{}^{Zr\text{-}L}$ (Zr_6O_6), but the atoms were allowed to move in any direction during. Details about the refinement in (c) and (d) are given below in S1.

Table S4: Refinement results parent UiO-66 (1-phase).

variable	formatted
R_w	0.30743167(0)
Reduced χ^2	0.1514883(0)
U_{iso} O (Å^2)	0.009(2)
U_{iso} Zr (Å^2)	0.0020(4)
Scale	0.41(2)

Table S5: Refinement results parent UiO-66, allowing isotropic cluster expansion/contraction using the *Zoomscale* parameter.

variable	formatted
R_w	0.27298469(0)
Reduced χ^2	0.11970959(0)
U_{iso} O (Å^2)	0.009(2)
U_{iso} Zr (Å^2)	0.0018(4)
Scale	0.40(2)
Zoomscale	0.996(1)

Table S6: Refinement results parent UiO-66. The DFT-optimized symmetric Zr_6O_8 was used as the starting model, but the atoms were allowed to move freely. A qualitative description of how the atoms moved during the refinement is given below in Section S1.

variable	formatted
R_w	0.22706165(0)
Reduced χ^2	0.07696662(0)
$_{iso}$ O (Å^2	0.0010000(6)
U_{iso} Zr0 (Å^2	0.0010000(3)
U_{iso} Zr1 (Å^2	0.0010000(3)
U_{iso} Zr2 (Å^2	0.001(3)
U_{iso} Zr3 (Å^2	0.001(3)
U_{iso} Zr4 (Å^2	0.001(5)
U_{iso} Zr5 (Å^2	0.001(4)
Scale	0.54(3)

Table S7: Refinement results UiO-66-RHT-40 (1-phase). The DFT-optimized dehydroxylated Zr_6O_6 structure was used as the starting model, but the atoms were allowed to move freely. A qualitative description of how the atoms moved during the refinement is given below in Section S1.

variable	formatted
R_w	0.23433087(0)
Reduced χ^2	0.05267705(0)
U_{iso} O (Å^2)	1.00(4)e-05
U_{iso} Zr0 (Å^2)	0.004(3)
U_{iso} Zr1 (Å^2)	0.001(2)
U_{iso} Zr2 (Å^2)	0.004(4)
U_{iso} Zr3 (Å^2)	0.000(3)
U_{iso} Zr4 (Å^2)	0.001(4)
U_{iso} Zr5 (Å^2)	0.002(5)
Scale	0.53(4)

S1 Refinement of atom positions Zr clusters

Analyzing how the structure of the dehydrated Zr_6O_6 cluster changes after refining the atom positions is not straightforward due to the low symmetry of the structure. The experimental PDF of the local structure in UiO-66 contains mainly signal coming from Zr-O bonds (first peak) and Zr-Zr pairs (second and third peak). Missing-linker defects are in the form of terminal OH^{-1}/H_2O groups.[3,4] These types of defects will affect the shape and broadness of the first-shell peak in the PDF. Cluster dehydroxylation, on the other hand, affects both the first shell peak and the peaks coming from Zr-Zr pairs.[2,5] Therefore, to understand how the cluster distorts during refinement, we compare the average Zr-O and Zr-Zr distances before and after refinement. In a conventional, high-symmetry pristine $\{Zr_6\}$, we would refer to the different oxygens as μ_3O, μ_3OH, and $O_{carboxy}$ atoms of carboxylate ligands, and the Zr-Zr distances can be grouped into shorter edge-sharing pairs and longer trans-positioned atoms. Because we allow the atoms to move and thus break the symmetry of the cluster, we group the different oxygens into bridging (Zr-O_{bridge}) and terminal (Zr-O_{term}) Zr-O bonds. In Figure 2 we show schematically what would be

included in Zr-O_{term} and Zr-O_{bridge} groups in a real sample. We also try to illustrate the effect of the symmetry breaking on the bridging oxygen groups. As a result of cluster dehydroxylation and distortion, the remaining Zr-O_{bridge} groups are no longer sitting in a high-symmetry octahedral site with equal Zr-O bond lengths. Instead, they have two shorter Zr-O distances and one longer (μ_2O character). This comes from the splitting of the edge-sharing Zr-Zr pairs into a longer and shorter Zr-Zr pair (Figure 1). We note that the conversion of μ_3O to μ_2O groups has also been described by Platero-Prats *et al.* with ab-initio molecular dynamics calculations to see how $\{Zr_6\}$ distort at elevated temperatures.[6]

S1.1 UiO after *RHT*

We first examine how the bond distances change as the atom positions of the DFT-optimized dehydrated cluster are refined to match the PDF of UiO-66-RHT-40. The average bond length for Zr-O_{term} changes from 2.20(2) to 2.19(13), while the Zr-O_{bridge} distances change from 2.049(9) to 2.10(5) from the DFT-optimized structure to the refined cluster from PDF analysis (Table S8). These differences are quite small, and the most noteworthy difference is the increased standard deviation (numbers in brackets) after refining the structures. This is due to further symmetry-breaking during the refinement of the atom positions to fit the experimental PDF. Bigger changes are observed for the Zr-Zr distances, which we group into Zr-Zr_{edge}^{short}, Zr-Zr_{edge}^{long}, and Zr-Zr_{trans} to account for the three Zr-Zr peaks in the experimental PDF after the *RHT* (Figure 1 c-d). The Zr-Zr_{edge}^{short} change from 3.414(0) to 3.460(96), the Zr-Zr_{edge}^{long} from 3.688(0) to 3.9(1), and the Zr-Zr_{trans} from 5.026(0) to 5.03(35) during refinement of the atom positions to the experimental PDF of UiO-66-RHT-40. We see that the largest changes are in the Zr-Zr_{edge}^{long} distances, which did not match the PDF very well in the DFT-optimized cluster before refinement.[2] Standard DFT calculations are done at 0 K, and effects of phonons are not accounted for. All bond distances are summarized in Table S8, and the structures before and after refinement can

be inspected visually in Figure S7. Since the structural and energetic differences between the DFT-optimized and refined dehydrated structures are relatively small, we use the refined structure as a representative model of the $\{Zr_6\}$ in UiO-66-RHT-40, as it matches the experimental PDF very well (Figure S6).

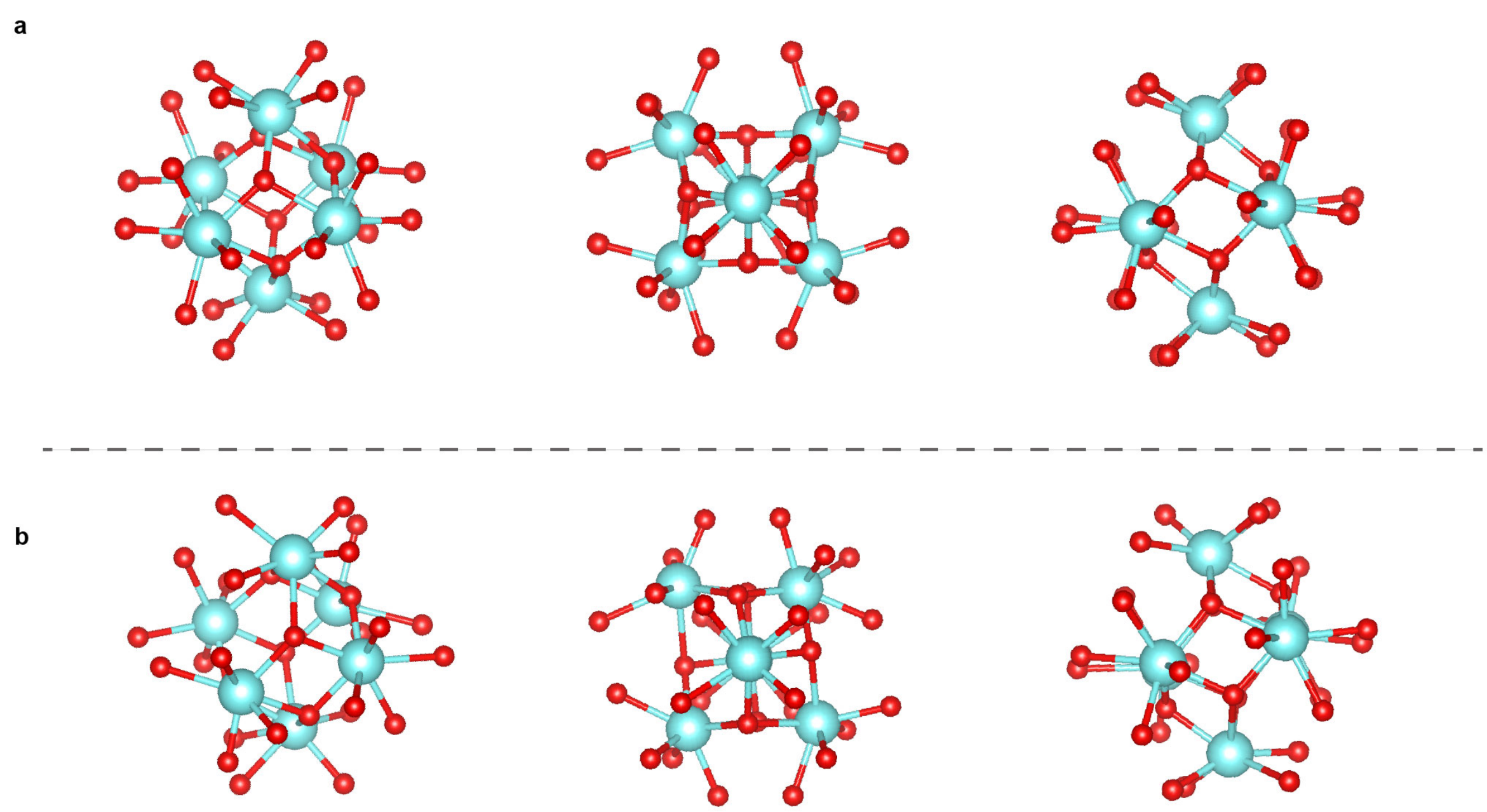

Figure S7: The DFT-optimized dehydrated cluster, after removing all H and C atoms, is shown on top (a) from different perspectives. At the bottom (b) is the cluster after refining the atom positions to improve the fit to the experimental PDF of UiO-66-RHT-40.

Table S8: The obtained Zr-Zr pairs and first shell Zr-O bond distances obtained from the DFT-optimized dehydrated cluster (DFT-opt) and from the subsequent refined structure after fitting to the experimental PDF of UiO-66-RHT-40 (refined, bottom).

DFT-opt	Zr-Zr_{short}	Zr-Zr_{long}	Zr-Zr_{trans}	Zr-μ_2O	Zr-O_{term}
average	3.414	3.688	5.026	2.050	2.2
st. dev.	0.0	0.0	0.0	0.009	0.017
MIN	3.414	3.688	5.026	2.04	2.183
MAX	3.414	3.688	5.026	2.058	2.226
refined	Zr-Zr_{short}	Zr-Zr_{long}	Zr-Zr_{trans}	Zr-μ_2O	Zr-O_{term}
average	3.46	3.9	5.03	2.10	2.19
st. dev.	0.096	0.1	0.35	0.05	0.13
MIN	3.344	3.744	4.73	1.973	1.958
MAX	3.607	3.959	5.418	2.173	2.407

S1.2 Parent UiO-66 (as-synthesized)

Interestingly, by comparing the structures before and after refinement to the parent UiO-66 (as-synthesized), we find that even the refinement of the hydrated Zr_6O_8 cluster to the parent UiO-66 led to symmetry breaking of the cluster, creating distortions that are similar, but less severe, to the dehydrated cluster (Figure S8). To improve the fit to the PDF, the edge-sharing Zr-Zr pairs are also split into shorter and longer pairs (Table S9). We do not consider the refined Zr_6O_8 cluster a realistic structural model for the $\{Zr_6\}$ in the parent UiO-66. However, the fact that the original high-symmetry cluster (Zr_6O_8) does not fit very well and lowers its symmetry to fit the measured PDF indicates that there are likely already some low-symmetry phases present in as-synthesized MOFs. In fact, the presence of distorted low-symmetry structures has been observed in PDFs of discrete benzoic acid-capped $\{Zr_6\}$ clusters.[6] It is quite likely that such low-symmetry clusters can be present in powders of UiO-66 as well.

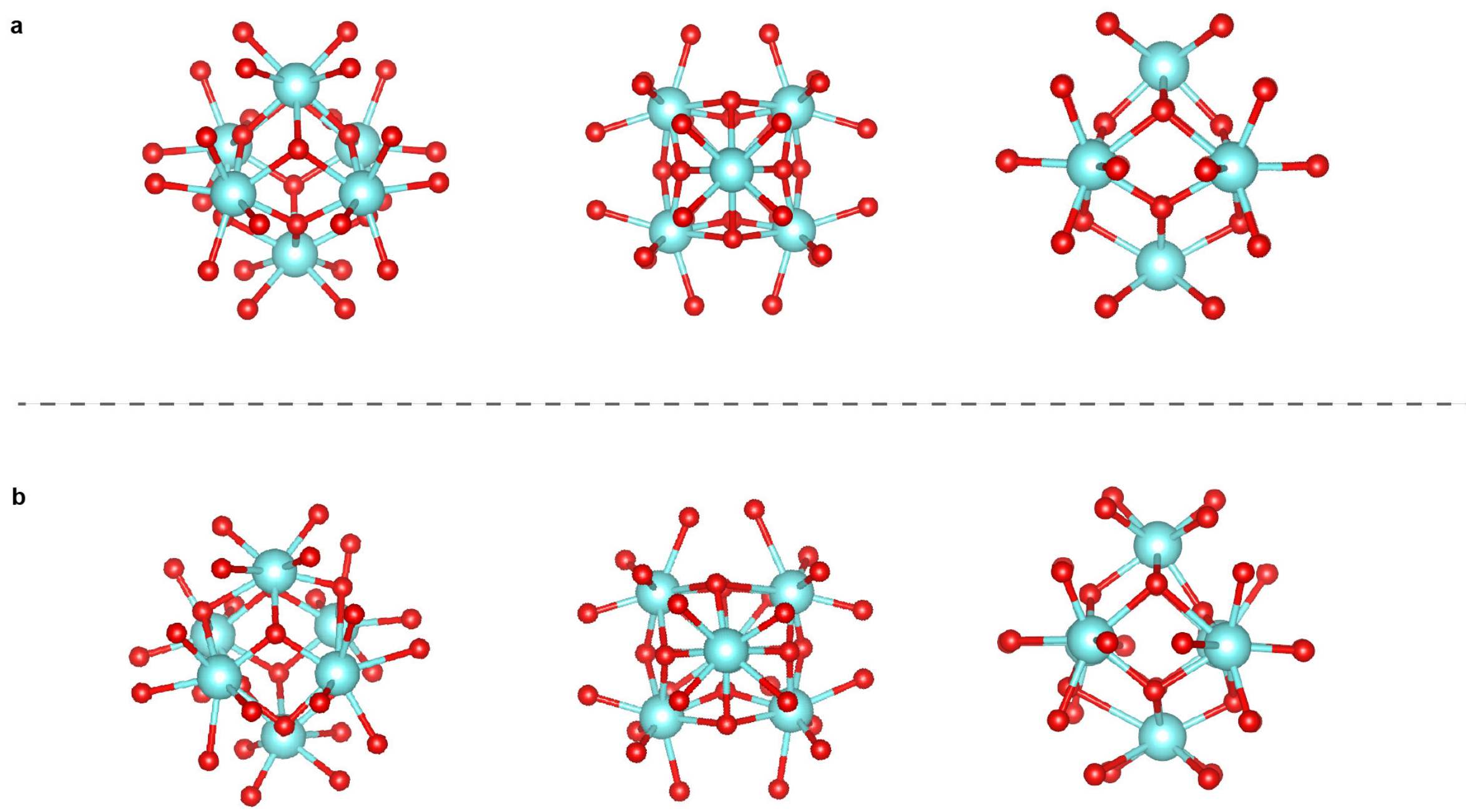

Figure S8: The DFT-optimized hydrated cluster, after removing all H and C atoms is shown on top (a) from different perspectives. At the bottom (b) is the cluster after refining the atom positions to improve the fit to the experimental PDF of the parent UiO-66. The distortion can be best seen in the view-point in the most-right of the Figure.

Table S9: The obtained Zr-Zr pair distances and first shell Zr-O bond distances obtained from the DFT-optimized hydrated cluster (DFT-opt) and from the subsequent refined structure after fitting to the experimental PDF of the parent UiO-66 (refined, bottom). Note that in this case, the Zr-O_{bridge} contains both μ_3O and μ_3OH groups. They are grouped together because their symmetry breaks during the refinement. The minimum and maximum values in that column for the DFT-optimized case correspond to Zr-μ_3O and Zr-μ_3OH bonds, respectively.

DFT-opt	Zr-Zr_{edge}	Zr-Zr_{trans}	Zr-O_{bridge}	Zr-O_{term}
average	3.533	4.996	2.17	2.222
st. dev.	0.0	0.0	0.10	0.0
MIN	3.533	4.996	2.07	2.222
MAX	3.533	4.996	2.265	2.222
refined	Zr-Zr_{edge}	Zr-Zr_{trans}	Zr-O_{bridge}	Zr-O_{term}
average	3.5	5.00	2.24	2.24
st. dev.	0.1	0.06	0.18	0.13
MIN	3.320	3.744	1.938	2.021
MAX	3.759	3.959	2.794	2.569

S2 Two-phase fitting results

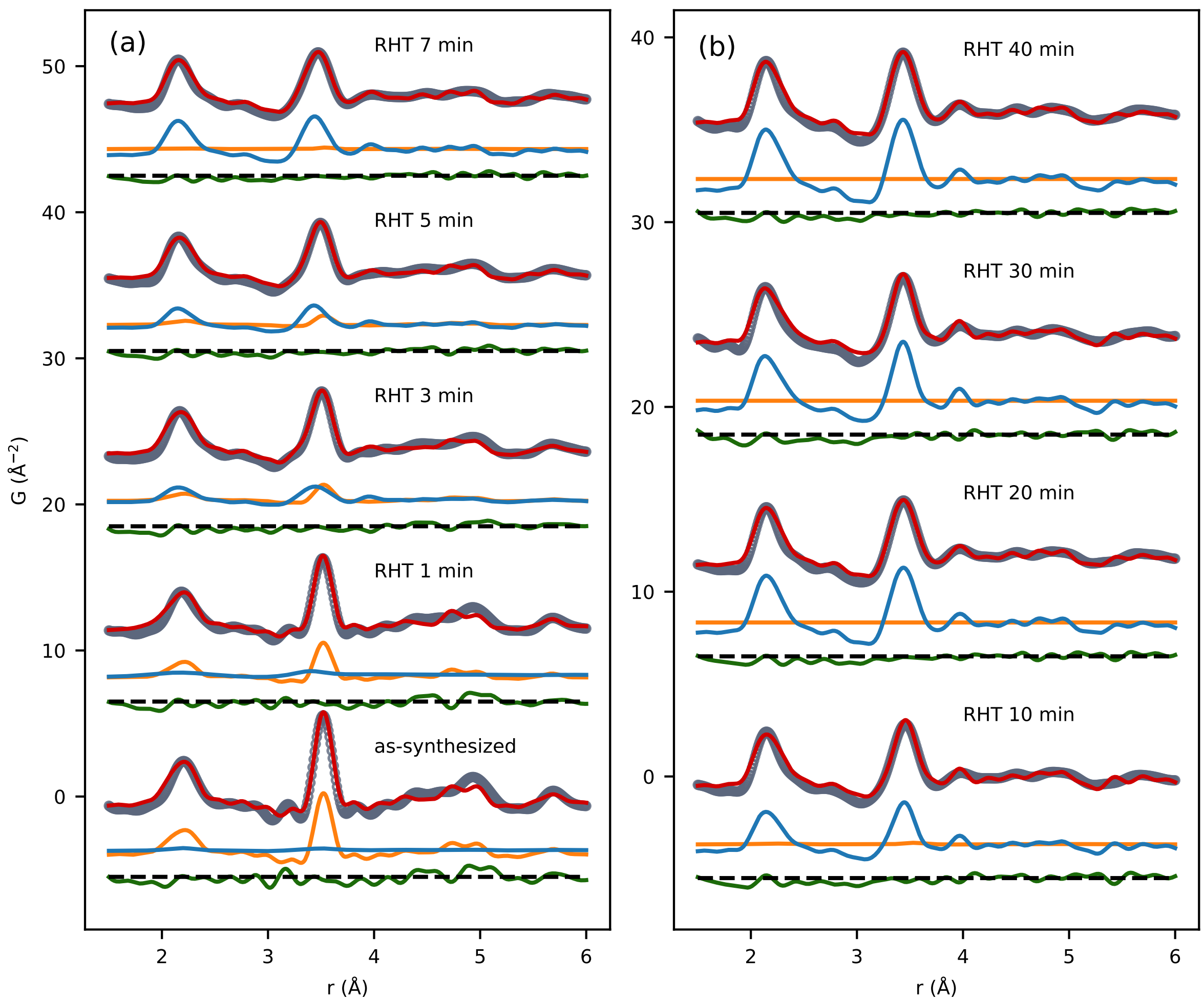

Figure S9: Two-phase model fit of the local structure in parent UiO-66 (as-synthesized) and samples exposed to the *RHT* for different times from 1-7 minutes (a) and 10-40 minutes (b). As phase 1, the DFT optimized Zr_6O_8 cluster, representing pristine and defect-free clusters, was used (orange). In phase 2, the DFT-optimized dehydrated cluster, where the Zr and O atom positions were further refined by fitting to the 40-minute sample, was used (blue). The linear combination of the two phases is plotted in red. Below each fit, the difference curve between the linear combination and the experimental data is plotted in green. The refined parameters are listed in Table S10.

Table S10: Refinement Results of two-phase model fits shown in Figure S9. G_1 refers to phase one (the hydrated Zr_6O_8 cluster, and G_2 refers to the dehydrated Zr_6O_6 cluster. The scaling factor s_1 refers to the mole fraction of G_1. The mole fraction of G_2 was constrained as $1 - s_1$. An overall scaling factor was used to scale the total PDF.

variable	parent UiO-66	RHT-1	RHT-3	RHT-5	RHT-7	RHT-10	RHT-20	RHT-30	RHT-40
Reduced χ^2	0.120069	0.077853	0.064669	0.045972	0.038701	0.047424	0.041696	0.05225	0.041515
R_w	0.274310	0.279847	0.260474	0.236385	0.220775	0.250299	0.223276	0.252803	0.208028
(G_1) Zoomscale	0.996(2)	0.995(3)	0.996(5)	0.996(7)	1.00(2)	1.00(2)	0(9)e+04	0(2)e+05	0(6)e+04
(G_1) O U_{iso} (Å^2)	0.010(4)	0.006(3)	0.009(8)	0.008(9)	0.01(2)	0.01(2)	0(3)e+05	0(3)e+05	0(1)e+05
(G_1) Zr1 U U_{iso} (Å^2)	0.0020(6)	0.0021(9)	0.003(1)	0.002(2)	0.002(4)	0.01(1)	0(3)e+04	0(5)e+04	0(2)e+04
(G_2) O U_{iso} (Å^2)	0.00(1)	0.03(6)	0.000(4)	1.00(5)e-05	0.000(3)	1.00(5)e-05	1.00(5)e-05	1.00(4)e-05	1.00(5)e-05
(G_2) Zr0 U_{iso} (Å^2)	0.1(6)	0.0(1)	0.004(9)	0.004(7)	0.005(5)	0.0001(5)	0.004(4)	0.000(2)	0.003(3)
(G_2) Zr1 U_{iso} (Å^2)	0.01(4)	0.0(2)	0.01(1)	0.001(4)	0.002(3)	0.003(4)	0.002(2)	0.002(2)	0.002(2)
(G_2) Zr2 U_{iso} (Å^2)	0.00(4)	0.0(3)	0.002(8)	0.003(6)	0.003(5)	0.004(5)	0.004(5)	0.005(5)	0.005(5)
(G_2) Zr3 U_{iso} (Å^2)	0.00(4)	0.1(4)	0.01(2)	0.002(6)	0.002(5)	0.000(3)	0.002(3)	0.002(3)	0.003(4)
(G_2) Zr4 U_{iso} (Å^2)	0.01(6)	0.0(2)	0.01(2)	0.01(1)	0.004(7)	0.003(9)	0.002(4)	0.002(6)	0.002(4)
(G_2) Zr5 U_{iso} (Å^2)	0.0(4)	0.1(6)	0.00(1)	0.000(5)	0.000(4)	0.005(8)	0.002(5)	0.01(1)	0.003(6)
Mole fraction G_1 (%)	0.7(2)	0.5(2)	0.4(1)	0.3(1)	0.11(9)	0.1(1)	-0(5)e-05	-0(4)e-05	-0(5)e-05
Mole fraction G_2 (%)	0.3(2)	0.5(2)	0.6(2)	0.7(1)	0.89(9)	0.9(1)	1.00000(5)	1.00000(4)	1.00000(5)
Overall scale	0.5(1)	0.5(2)	0.49(4)	0.45(4)	0.49(4)	0.48(4)	0.51(5)	0.50(4)	0.56(4)

S6 Arsenic adsorption kinetics

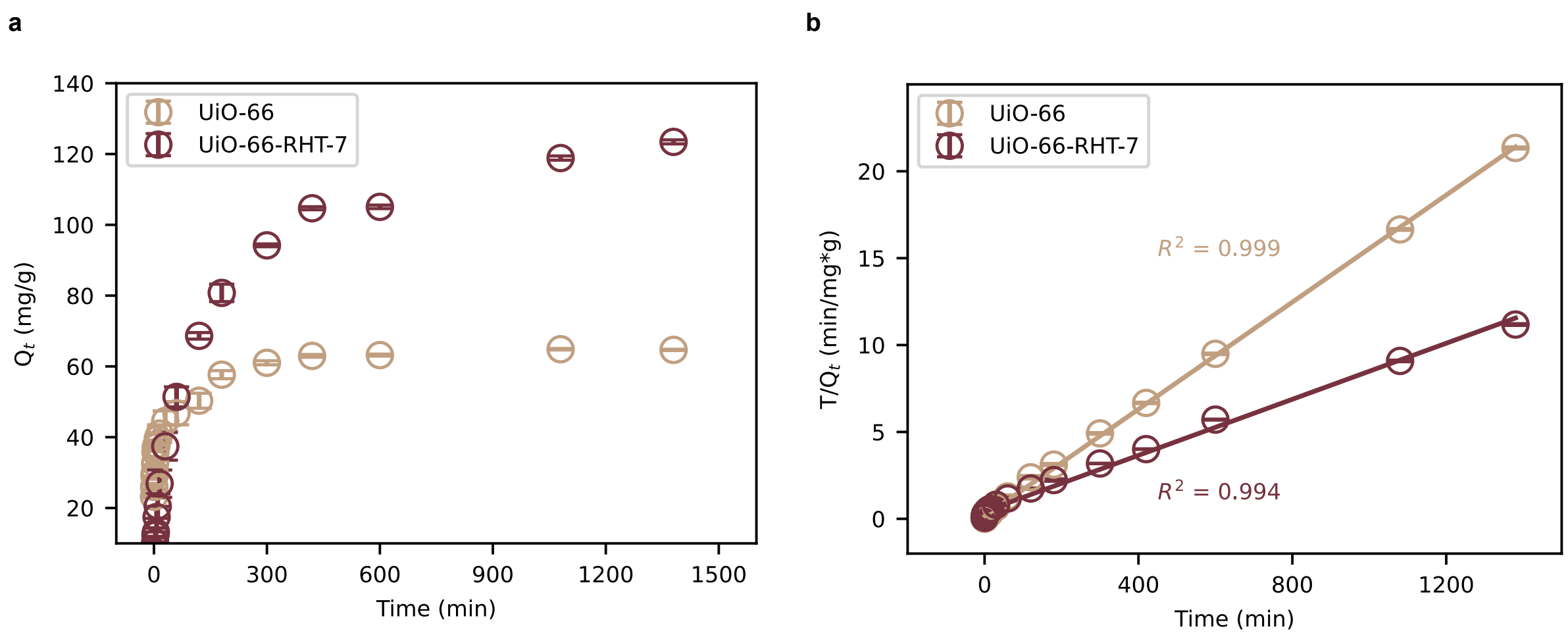

Figure S10: a) Kinetic adsorption experiment for the parent UiO-66 and UiO-66-RHT-7. b) linearized fit of pseudo-second order model (PSO).

Table S11: Parameters obtained by fitting the linearized pseudo-second-order model (PSO).

material	k_2	Q_e	R^2
Parent UiO-66	1.65×10^{-3}	64.89	0.99
UiO-66-RHT-7	1.51×10^{-4}	124.06	0.993

S7 Characterization of mixed linker MOFs (Method 2)

S1 Linker fraction analysis in mixed linker MOF using TGA

The relative content of BDC and BDC-NH_2 linkers was estimated from TGA. The decomposition temperatures of the individual linkers were identified by comparing the derivative thermogravimetric curves of UiO-66 and UiO-66-NH_2 (Figure S11a). BDC-NH_2 starts to decompose at approximately 325 °C, whereas BDC degradation begins at around 490 °C. Accordingly, the total organic linker content was assumed to correspond to the mass loss occurring between 340 and 800 °C.

In a first step, all mixed-linker samples were normalized to 100 % at 490 °C. The mass fractions at 340 °C, W_{340} (%), and at 800 °C, W_{800} (%), were extracted from the normalized TGA curves. The mass contributions of the two linkers were then approximated as

$$m_{\mathrm{BDC}\text{-}NH_2} = W_{340} - 100\%, \tag{4}$$

$$m_{\mathrm{BDC}} = W_{340} - W_{800}. \tag{5}$$

The fraction of BDC-NH_2 relative to the total linker content was calculated as

$$f_{\mathrm{BDC}\text{-}NH_2} = \frac{m_{BDC\text{-}NH_2}}{m_{BDC\text{-}NH_2} + m_{\mathrm{BDC}}} \tag{6}$$

TGA data of all samples and the corresponding calculated BDC-NH_2 fractions can be found in Figure S11 to S13 and Table S12, respectively. The mixed linker samples are named according to their calculated BDC-NH_2 content as ml-X, with X is the calculated fraction of BDC-NH_2.

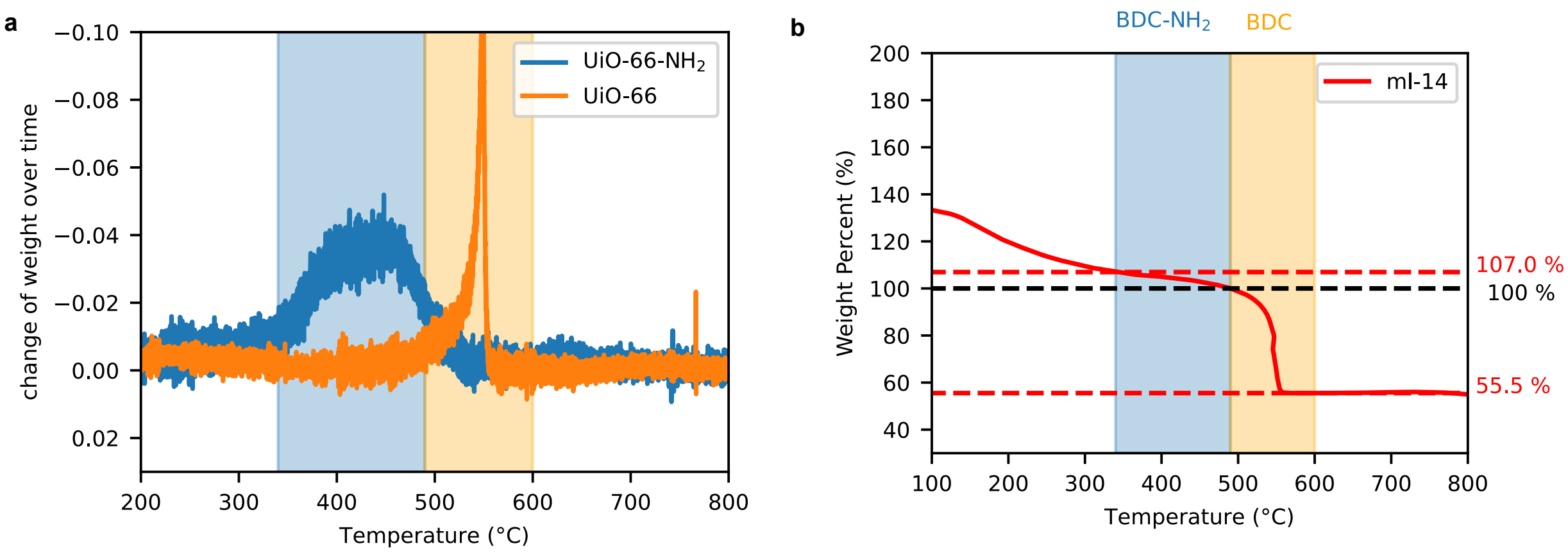

Figure S11: TGA for mixed linker MOFs: a) shows the derivative thermogravimetric curve for UiO-66 and UiO-66-NH_2. b) TGA curves and calculation of BDC and BDC-NH_2 linker content of mixed linker MOF from synthesis ratio of 5% BDC-NH_2

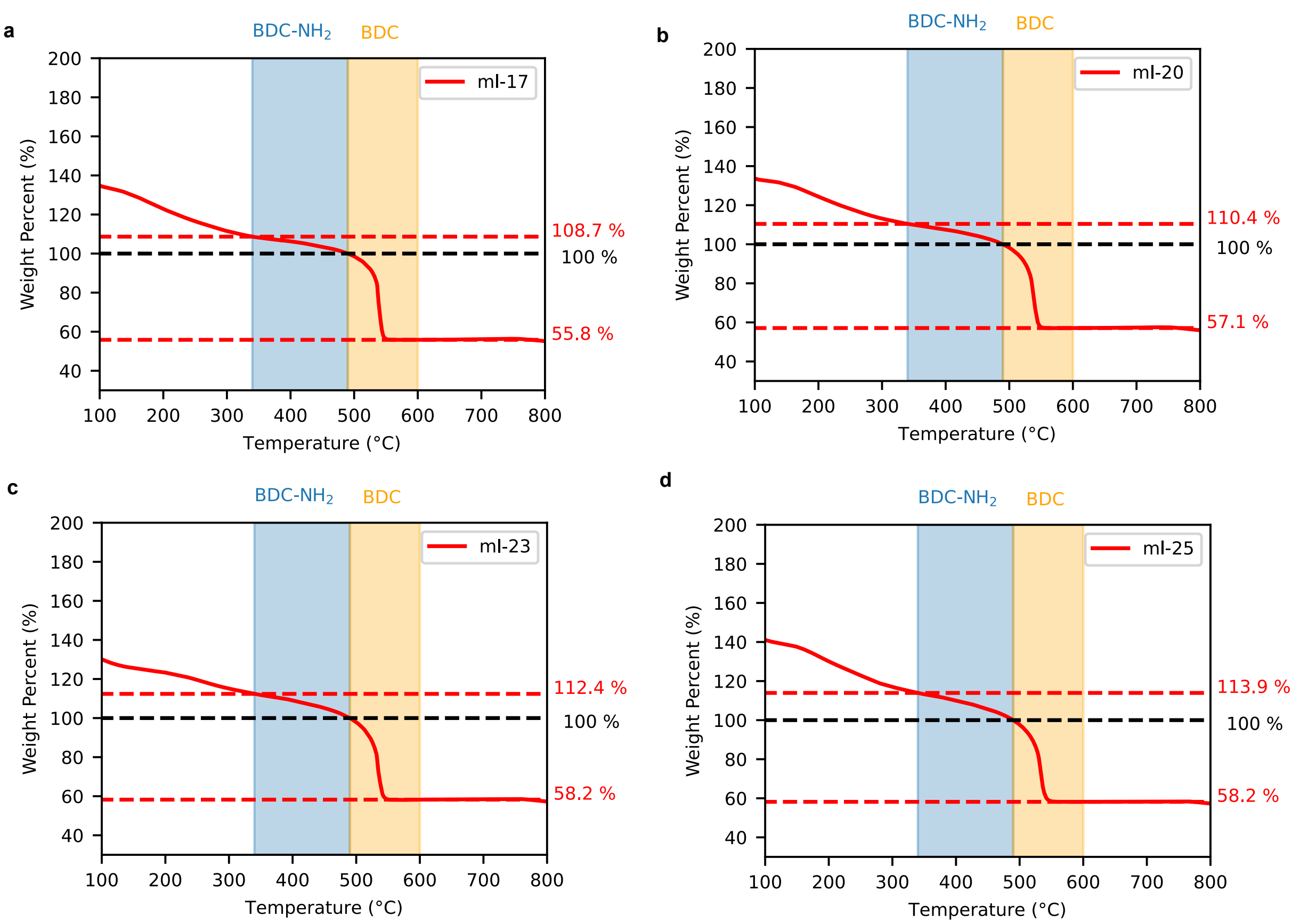

Figure S12: TGA curves and calculation of BDC and BDC-NH_2 linker content of mixed linker MOF from different synthesis ratio of a) 10% BDC-NH_2), b) 15% BDC-NH_2), c) 20% BDC-NH_2), d) 25% BDC-NH_2).

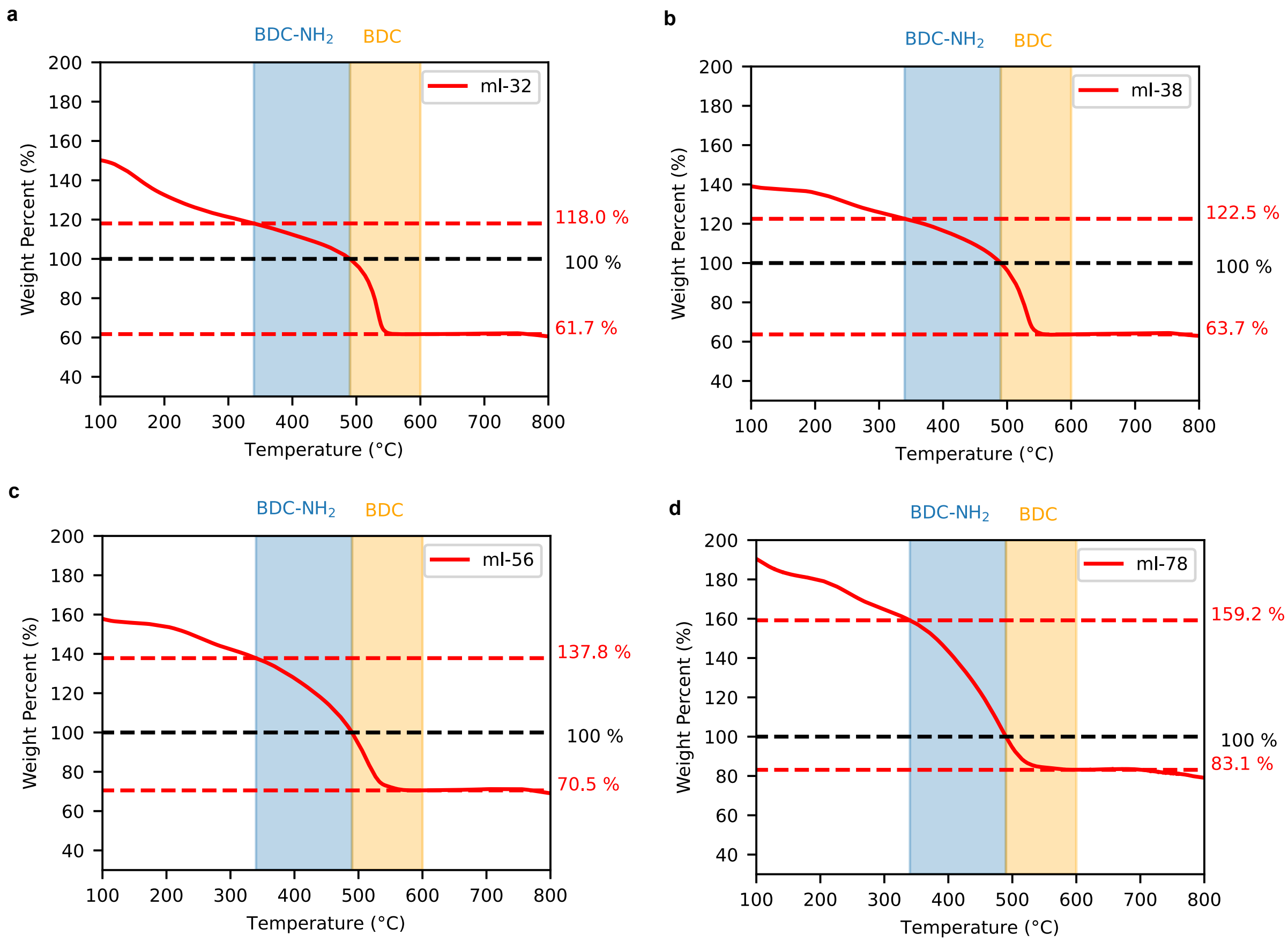

Figure S13: TGA curves and calculation of BDC and BDC-NH_2 linker content of mixed linker MOF from different synthesis ratio of a) 30% BDC-NH_2), b) 40% BDC-NH_2), c) 60% BDC-NH_2), d) 80% BDC-NH_2)

Table S12: Overview of the theoretical BDC-to-BDC-NH_2 ratios used in the synthesis, alongside the corresponding experimental ratios determined using TGA.

	Synthesis Ratio		TGA Analysis		
**Sample**	**BDC**	**BDC-NH_2**	**BDC (wt. %)**	**BDC-NH_2 (wt. %)**	**BDC-NH_2 (%)**
UiO-66	1.00	0	-	0	100
ml-14	0.95	0.05	44.5	7.0	$\approx$ 14
ml-17	0.90	0.10	44.2	8.7	$\approx$ 17
ml-20	0.85	0.15	42.9	10.4	$\approx$ 20
ml-23	0.80	0.20	41.8	12.4	$\approx$ 23
ml-25	0.75	0.25	41.8	13.9	$\approx$ 25
ml-32	0.70	0.30	38.3	18.0	$\approx$ 32
ml-38	0.60	0.40	36.3	22.5	$\approx$ 38
ml-56	0.40	0.60	29.5	36.8	$\approx$ 56
ml-78	0.20	0.80	16.9	59.2	$\approx$ 78
UiO-66-NH_2	0.00	1.00	0	-	100.0

S2 PXRD and N_2 adsorption isotherm

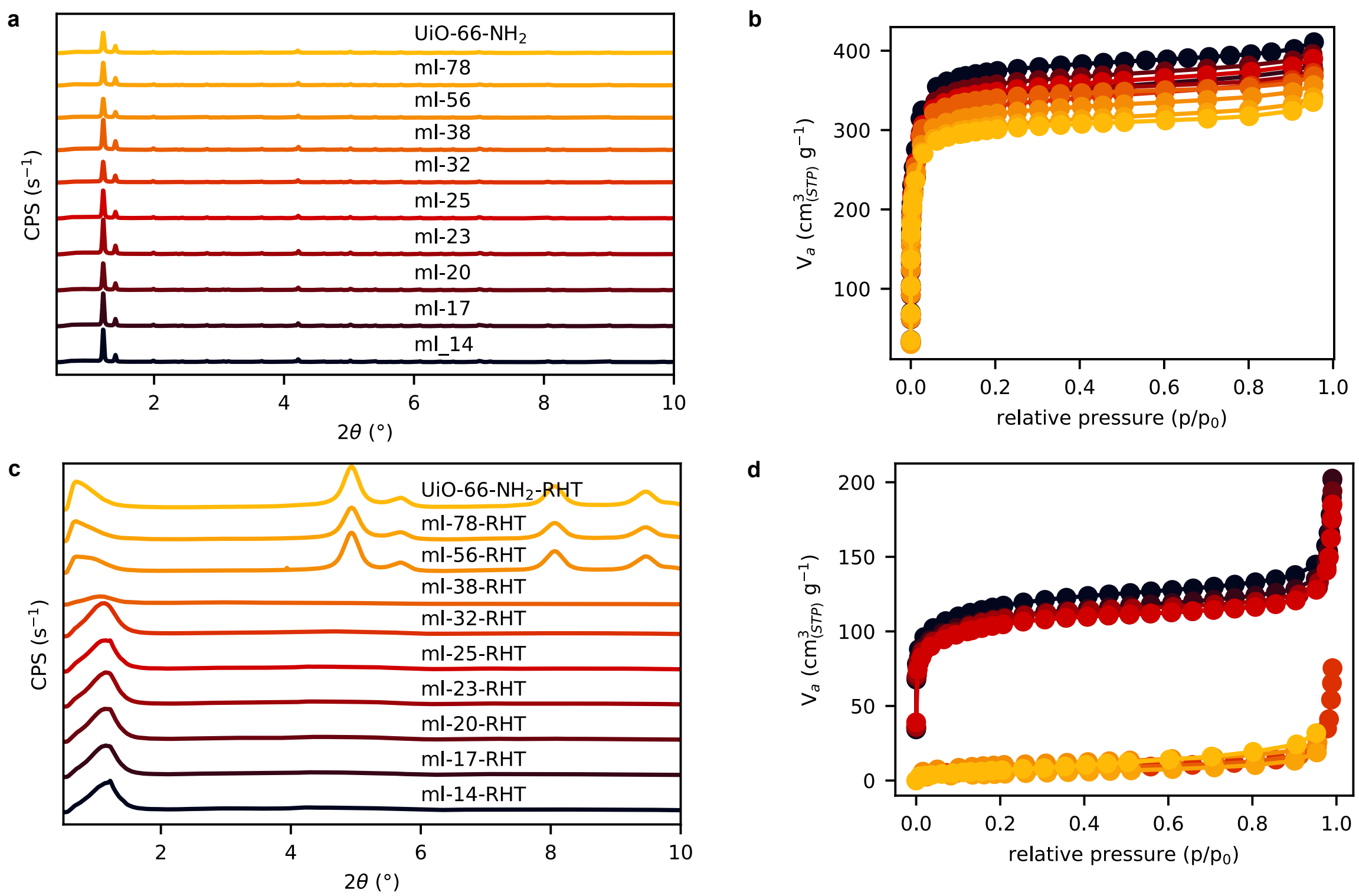

Figure S14: Overview mixed linker MOF (ml-MOF) and samples exposed to the *RHT* (ml-MOF-RHT). a) PXRD and b) N_2 adsorption isotherm for as synthesized ml-MOF. c) PXRD and d) N_2 adsorption isotherms for ml-MOF-RHT

Table S13: Brunauer–Emmett–Teller (BET) area of as-synthesized ml-MOFs, obtained using BETSI[1]

material (BDC-NH_2 content)	BET ($m^2\ g^{-1}$)
ml-14	1451
ml-17	1351
ml-20	1366
ml-23	1342
ml-25	1345
ml-32	1277
ml-38	1327
ml-56	1249
ml-78	1199
UiO-66-NH_2	1181

S3 TGA of mixed linker (BDC/BDC-NH_2) MOFs after *RHT*

Figure S15 to S17 show comparison of TGA data of mixed linker MOF and the *RHT* sample. Missing linkers were calculated the same way (see Equations (1) and (2) as described above for UiO-66. The results are summarized in Figure S17b and Table S14. The Zr content of the materials was calculated in the same way (see Equation (3)) as described above for UiO-66. The results are summarized in Table S15.

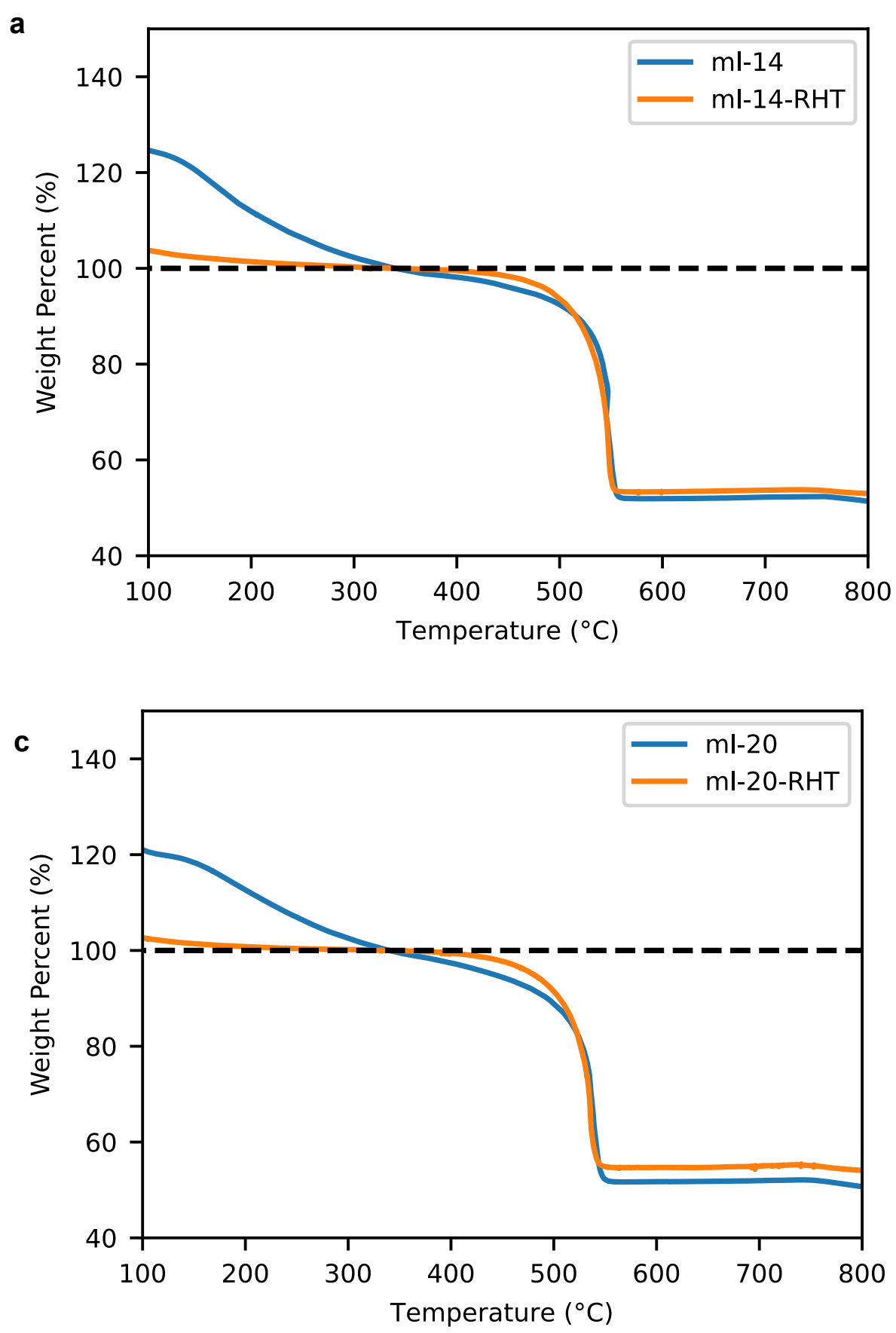

Figure S15: TGA comparison of as synthesized mixed linker MOFs and corresponding *RHT* treated sample for BDC-NH_2 content of a) 14% , b) 17% , c) 20% , d) 23%

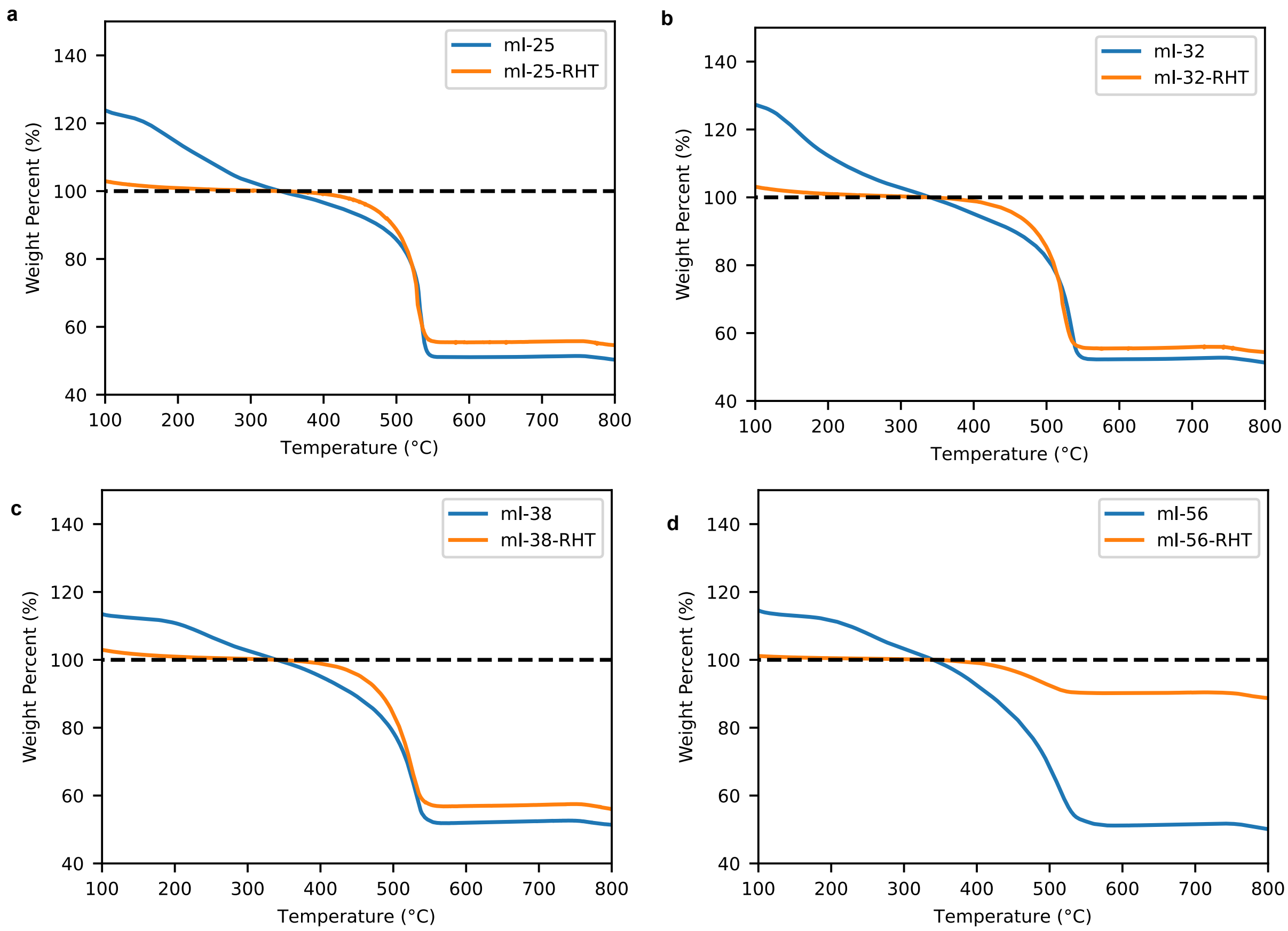

Figure S16: TGA comparison of as synthesized mixed linker MOFs and corresponding *RHT* treated sample for BDC-NH_2 content of a) 25%, b) 32% , c) 38% , d) 56%

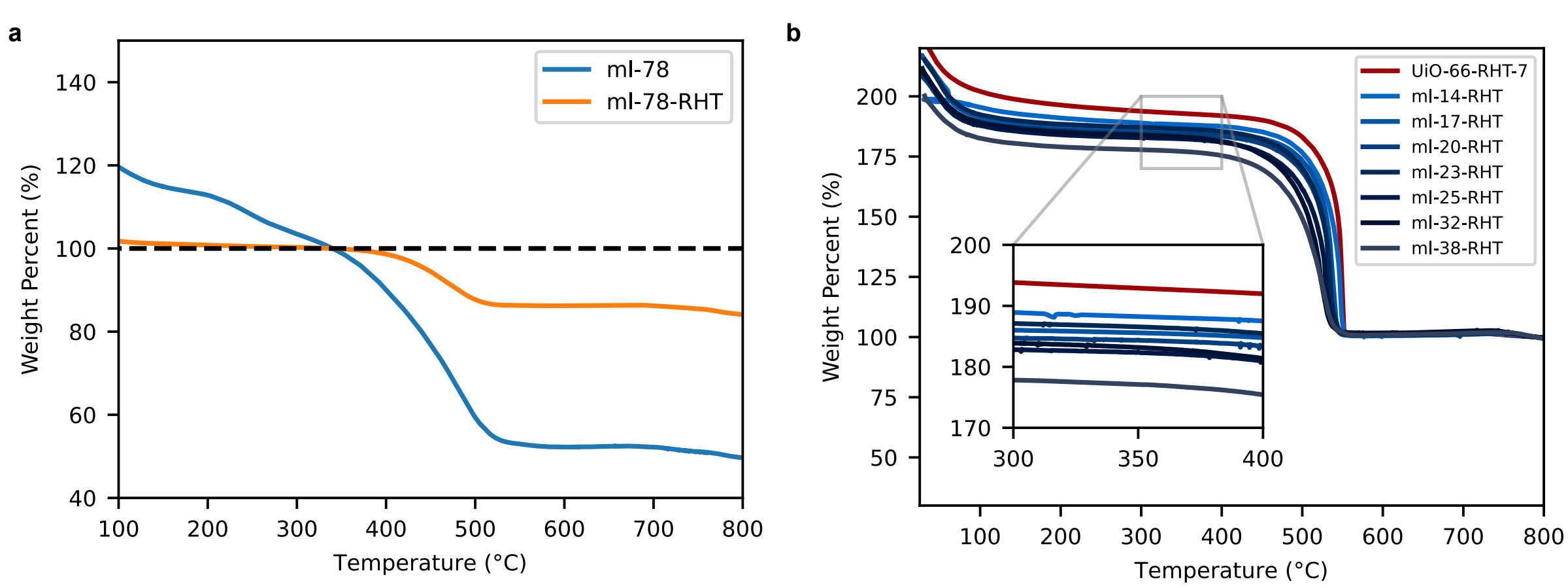

Figure S17: a) TGA comparison of as synthesized mixed linker MOFs and corresponding *RHT* treated sample for BDC-NH_2 content of 78%, b) comparison of all TGA profiles of *RHT* samples set to 100% at 800 °C.

Table S14: Overview of missing linkers determined by TGA.

material	wt. % norm to ZrO_2	Theoretical structure at 400 °C	Missing linker
UiO-66-RHT-7	192.22	$Zr_6O_6(O)_{1.40}(C_8H_6O_4)_{4.60}$	1.40
ml-14-RHT	187.35	$Zr_6O_6(O)_{1.64}(C_8H_6O_4)_{4.36}$	1.64
ml-17-RHT	185.51	$Zr_6O_6(O)_{1.73}(C_8H_6O_4)_{4.27}$	1.73
ml-20-RHT	183.72	$Zr_6O_6(O)_{1.82}(C_8H_6O_4)_{4.18}$	1.82
ml-23-RHT	186.10	$Zr_6O_6(O)_{1.70}(C_8H_6O_4)_{4.30}$	1.70
ml-25-RHT	181.63	$Zr_6O_6(O)_{1.93}(C_8H_6O_4)_{4.07}$	1.93
ml-32-RHT	181.79	$Zr_6O_6(O)_{1.92}(C_8H_6O_4)_{4.08}$	1.92
ml-38-RHT	176.37	$Zr_6O_6(O)_{2.19}(C_8H_6O_4)_{3.81}$	2.19
ml-56-RHT	111.69	$Zr_6O_6(O)_{5.42}(C_8H_6O_4)_{0.58}$	5.42
ml-78-RHT	117.25	$Zr_6O_6(O)_{5.14}(C_8H_6O_4)_{0.86}$	5.14
UiO-66-NH_2-RHT	109.17	$Zr_6O_6(O)_{5.54}(C_8H_5NO_4)_{0.46}$	5.54

Table S15: Overview Zr content

material	ZrO_2 wt. % at 800 °C	Zr content in wt. %
ml-14-RHT	46.30	34.26
ml-17-RHT	46.16	34.16
ml-20-RHT	48.09	35.58
ml-23-RHT	46.30	34.26
ml-25-RHT	47.41	35.08
ml-32-RHT	47.62	35.24
ml-38-RHT	49.68	36.76
ml-56-RHT	85.63	63.37
ml-78-RHT	80.07	59.25
UiO-66-NH_2-RHT	87.70	64.89

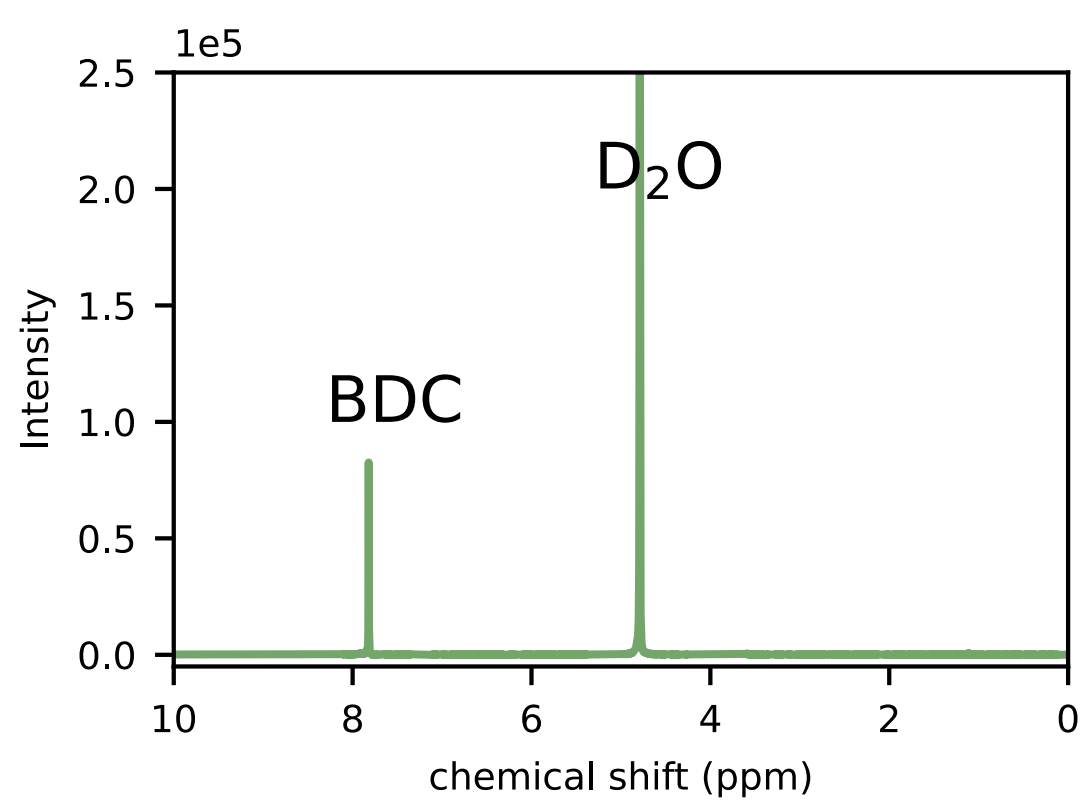

Figure S18: ^{1}H NMR of digested RHT 23 % BDC-NH_2 (ml-23-RHT)

S4 PDF analysis of ml-RHT series

Table S16: Combined Refinement Results Mixed linker MOFs after *RHT*. G_1 refers to phase one (the hydrated Zr_6O_8 cluster, and G_2 refers to the dehydrated Zr_6O_6 cluster. The scaling factor s_1 refers to the mole fraction of G_1. The mole fraction of G_2 was constrained as $1 - s_1$. An overall scaling factor was used to scale the total PDF.

variable	ml-14 RHT 2-phase	ml-17 RHT 2-phase	ml-20 RHT 2-phase	ml-23 RHT 2-phase	ml-25 RHT 2-phase	ml-32 RHT 2-phase	ml-38 RHT 2-phase	ml-56 RHT 2-phase	ml-78 RHT 2-phase
Reduced χ^2	0.046524	0.04371	0.045686	0.045685	0.048238	0.04969	0.056105	1.67653	3.68045
R_w	0.218764	0.208736	0.212634	0.215682	0.216727	0.217959	0.22732	0.410405	0.647618
(G_1) Zoomscale	1.00(3)	1.00(2)	0.99(2)	1.00(2)	1.00(4)	1.00(5)	0(1)e+05	1.018(1)	0.999(4)
(G_1) O_1 U_{iso} (Å^2)	0.01(3)	0.01(3)	0.01(2)	0.01(3)	0.01(5)	0.0(2)	0(2)e+05	0.09(1)	0.02(1)
(G_1) Zr_1 U_{iso} (Å^2)	0.00(1)	0.001(4)	0.000(3)	0.001(5)	0.00(1)	0.000(9)	0(4)e+04	0.0040(4)	0.005(2)
O2 U_{iso} (Å^2)	0.000(2)	1.00(5)e-05	1.00(5)e-05	1.00(5)e-05	1.00(5)e-05	0.000(2)	1.00(4)e-05	1.000(8)e-05	0.03(2)
(G_2) Zr_0 U_{iso} (Å^2)	0.005(4)	0.005(5)	0.005(5)	0.005(5)	0.005(5)	0.005(4)	0.005(4)	0.02(3)	0.01(1)
(G_2) Zr_1 U_{iso} (Å^2)	0.002(3)	0.001(3)	0.001(3)	0.001(3)	0.002(3)	0.002(2)	0.001(2)	0.00010000(8)	0.01(2)
(G_2) Zr_2 U_{iso} (Å^2)	0.003(4)	0.003(4)	0.003(4)	0.003(4)	0.003(3)	0.004(4)	0.003(3)	0.000(3)	0.002(7)
(G_2) Zr_3 U_{iso} (Å^2)	0.001(3)	0.003(5)	0.002(5)	0.002(5)	0.001(4)	0.001(4)	0.003(3)	0.000(4)	0.00(1)
(G_2) Zr_4 U_{iso} (Å^2)	0.003(4)	0.004(6)	0.004(6)	0.003(5)	0.003(5)	0.003(5)	0.002(3)	0.01(1)	0.01(3)
(G_2) Zr_5 U_{iso} (Å^2)	0.001(4)	0.000(3)	0.000(4)	0.001(4)	0.001(4)	0.001(3)	0.001(3)	0.000(4)	0.01(4)
Mole fraction G_1 (%)	0.1(1)	0.06(7)	0.06(6)	0.06(7)	0.03(7)	0.02(6)	-0(4)e-05	0.83(3)	0.54(9)
Mole fraction G_2 (%)	0.9(1)	0.94(7)	0.94(6)	0.94(7)	0.97(7)	0.98(6)	1.00000(4)	0.18(3)	0.46(9)
Overall scale	0.54(4)	0.56(4)	0.56(4)	0.55(4)	0.56(4)	0.57(4)	0.58(4)	1.478(5)	1.02(5)

S8 Characterization of base treated MOFs (Method 3)

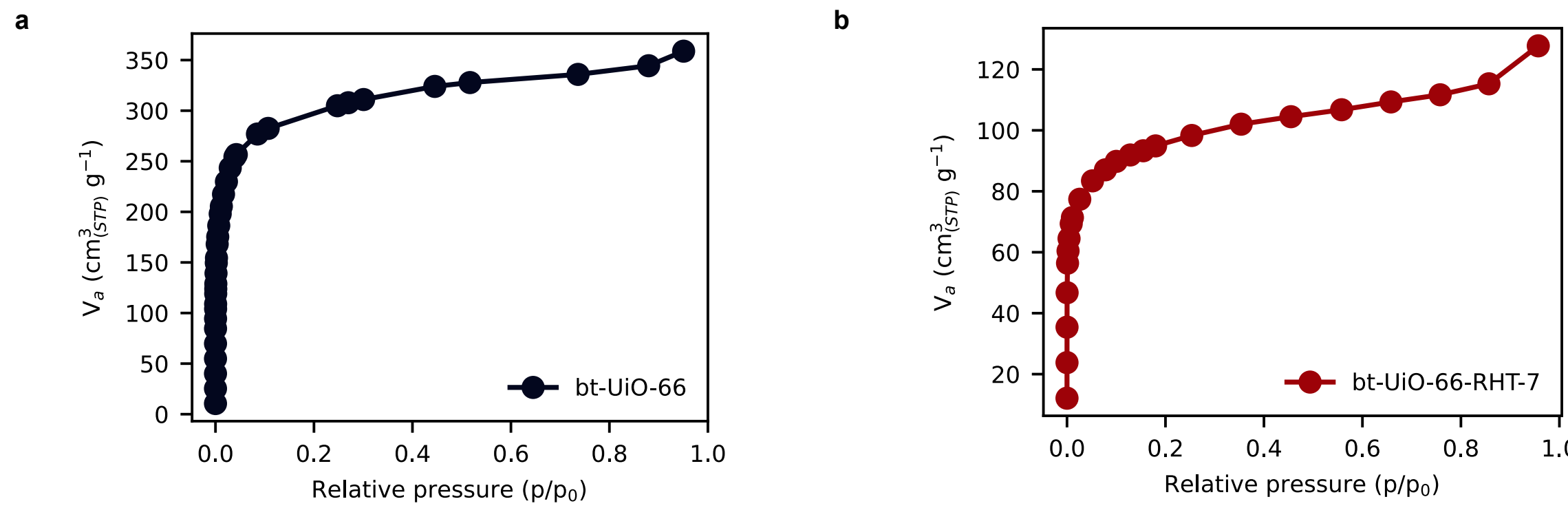

Figure S19: N_2 adsorption isotherm for a) bt-UiO-66 and b) bt-UiO-66-RHT-7.

S1 TGA of base-treated UiO-66 (bt-UiO-66) and UiO-66-*RHT*-7

Figure S20 show comparison of TGA data of as-synt UiO-66 and UiO-66-RHT-7 MOF before and after base treatment. Missing linkers were calculated the same way (see Equations (1) and (2) as described above for UiO-66. The results are summarized in Table S17. The Zr content of the materials was calculated in the same way (see Equation (3)) as described above for UiO-66. The results are summarized in Table S18.

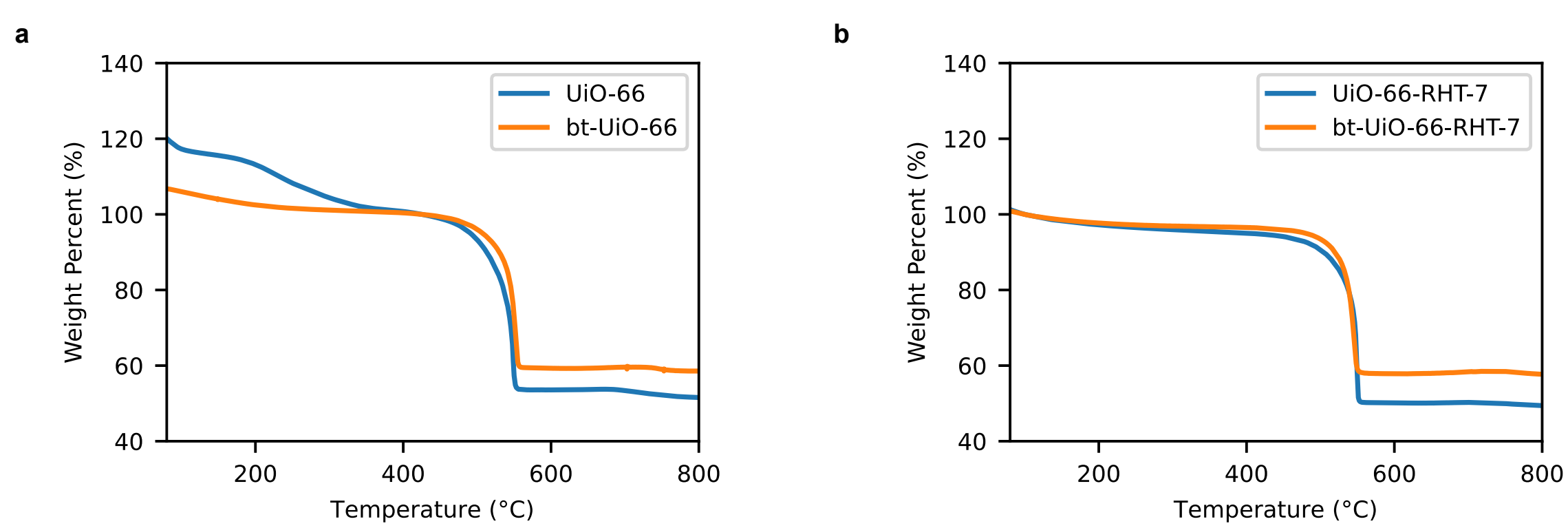

Figure S20: TGA profiles of a) UiO-66 and b) UiO-66-RHT-7 and corresponding base treated sample.

Table S17: Overview of missing linkers determined by TGA.

**material**	**wt. % norm to ZrO_2**	**Theoretical structure**	**Missing linker**
UiO-66	195.34	$Zr_6O_6(O)_{1.24}(C_8H_6O_4)_{4.76}$	1.24
bt-UiO-66	171.43	$Zr_6O_4(O)_{2.43}(C_8H_6O_4)_{3.57}$	2.43
UiO-66-RHT-7	192.22	$Zr_6O_6(O)_{1.40}(C_8H_6O_4)_{4.60}$	1.40
bt-UiO-66-RHT-7	167.18	$Zr_6O_6(O)_{2.65}(C_8H_6O_4)_{3.35}$	2.65

Table S18: Overview Zr content

**material**	**ZrO_2 wt. %** at 800 °C	**Zr content in wt. %**
UiO-66	36.53	27.03
bt-UiO-66	45.22	33.46
UiO-66-RHT-7	44.68	33.07
bt-UiO-66-RHT-7	50.40	37.30

S2 PDF analysis of base treated MOFs

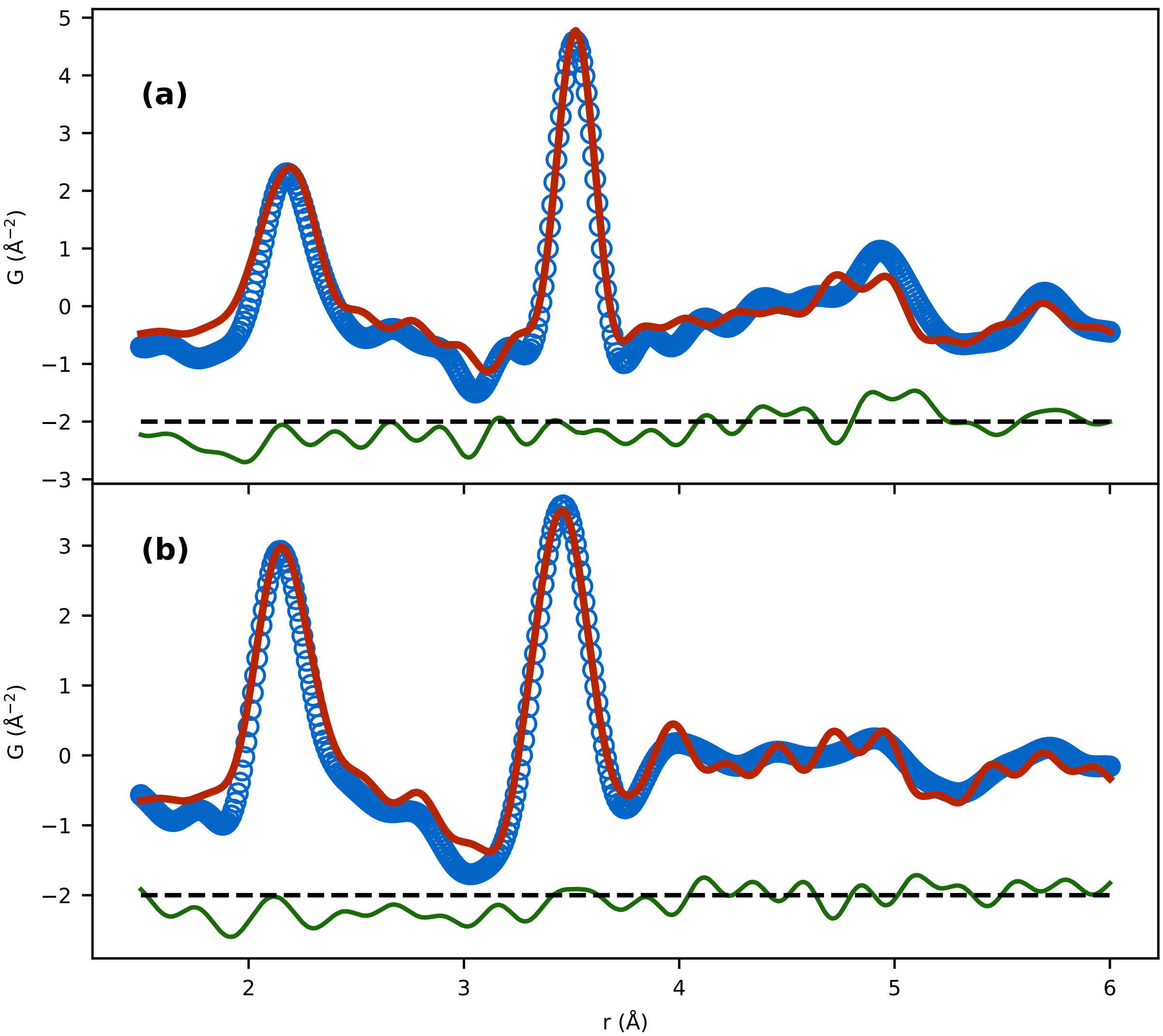

Figure S21: Two-phase model fit of the local structure in bt-UiO-66 (a) and bt-UiO-66-RHT-7 (b). As phase 1, the DFT optimized Zr_6O_8 cluster, representing pristine and defect-free clusters, was used. In phase 2, the DFT-optimized dehydrated cluster, where the Zr and O atom positions were further refined by fitting to the 40-minute sample, was used. The linear combination of the two phases is plotted in red. Below the fit, the difference curve between the linear combination and the experimental data is plotted in green.

Table S19: Refinement Results for based treated samples (parent MOF and UiO-66-RHT-7, respectively). G_1 refers to phase one (the hydrated Zr_6O_8 cluster, and G_2 refers to the dehydrated Zr_6O_6 cluster. The scaling factor s_1 refers to the mole fraction of G_1. The mole fraction of G_2 was constrained as $1 - s_1$. An overall scaling factor was used to scale the total PDF.

variable	bt-Uio-66 2-phase	bt-Uio-66-RHT-7 2-phase
Reduced χ^2	0.086934	0.051561
R_w	0.273974	0.211499
(G_1) Zoomscale	0.996(4)	1.00(3)
(G_1) O1 U_{iso} (Å^2)	0.009(6)	0.01(5)
(G_1) Zr1 U_{iso} (Å^2)	0.002(1)	0.00(1)
(G_2) O2 U_{iso} (Å^2)	0.000(6)	1.00(4)e-05
(G_2) Zr0 U_{iso} (Å^2)	0.01(1)	0.005(4)
(G_2) Zr1 U_{iso} (Å^2)	0.01(2)	0.002(2)
(G_2) Zr2 U_{iso} (Å^2)	0.001(7)	0.003(3)
(G_2) Zr3 U_{iso} (Å^2)	0.00(1)	0.001(3)
(G_2) Zr4 U_{iso} (Å^2)	0.01(3)	0.002(3)
(G_2) Zr5 U_{iso} (Å^2)	0.000(9)	0.001(3)
Mole fraction G_1 (%)	0.5(1)	0.1(1)
Mole fraction G_2 (%)	0.5(1)	0.9(1)
Overall scale	0.47(4)	0.59(4)

References

(1) Osterrieth, J. W. M. et al. How Reproducible Are Surface Areas Calculated from the BET Equation? *Advanced Materials* **2022**, *34*, 2201502.

(2) Schertenleib, T.; Asgari, M.; Mouriño, B.; Karve, V. V.; Felder, T. M. O.; Stoian, D.; Bon, V.; Hao, J.; Ortega-Guerrero, A.; Oveisi, E.; Agrawal, K. V.; Smit, B.; Kaskel, S.; Billinge, S. J. L.; Queen, W. L. Anisotropic Node Distortions in Amorphous MOFs: Low-valent Zr Sites as Catalytic Hotspots. *Chem* **2025**, *11*, 102619.

(3) Trickett, C. A.; Gagnon, K. J.; Lee, S.; Gándara, F.; Bürgi, H.-B.; Yaghi, O. M. Definitive Molecular Level Characterization of Defects in UiO-66 Crystals. *Angewandte Chemie International Edition* **2015**, *54*, 11162–11167.

(4) Øien, S.; Wragg, D.; Reinsch, H.; Svelle, S.; Bordiga, S.; Lamberti, C.; Lillerud, K. P. Detailed Structure Analysis of Atomic Positions and Defects in Zirconium Metal–Organic Frameworks. *Crystal Growth & Design* **2014**, *14*, 5370–5372.

(5) Valenzano, L.; Civalleri, B.; Chavan, S.; Bordiga, S.; Nilsen, M. H.; Jakobsen, S.; Lillerud, K. P.; Lamberti, C. Disclosing the Complex Structure of UiO-66 Metal Organic Framework: A Synergic Combination of Experiment and Theory. *Chemistry of Materials* **2011**, *23*, 1700–1718.

(6) Platero-Prats, A. E.; Mavrandonakis, A.; Gallington, L. C.; Liu, Y.; Hupp, J. T.; Farha, O. K.; Cramer, C. J.; Chapman, K. W. Structural Transitions of the Metal-Oxide Nodes within Metal–Organic Frameworks: On the Local Structures of NU-1000 and UiO-66. *Journal of the American Chemical Society* **2016**, *138*, 4178–4185.
